# Association of Perioperative Skeletal Muscle Index Change With Outcome in Colorectal Cancer Patients

**DOI:** 10.1002/jcsm.13594

**Published:** 2024-10-03

**Authors:** Zhenhui Li, Guanghong Yan, Mengmei Liu, Yanli Li, Lizhu Liu, Ruimin You, Xianshuo Cheng, Caixia Zhang, Qingwan Li, Zhaojuan Jiang, Jinqiu Ruan, Yingying Ding, Wenliang Li, Dingyun You, Zaiyi Liu

**Affiliations:** ^1^ Guangdong Cardiovascular Institute Guangdong Provincial People's Hospital, Guangdong Academy of Sciences Guangzhou China; ^2^ Department of Radiology, Guangdong Provincial People's Hospital (Guangdong Academy of Medical Sciences) Southern Medical University Guangzhou China; ^3^ Guangdong Provincial Key Laboratory of Artificial Intelligence in Medical Image Analysis and Application Guangzhou China; ^4^ Department of Radiology The Third Affiliated Hospital of Kunming Medical University, Yunnan Cancer Hospital, Yunnan Cancer Center Kunming China; ^5^ Yunnan Provincial Key Laboratory of Public Health and Biosafety Kunming Medical University Kunming China; ^6^ Department of Colorectal Surgery The Third Affiliated Hospital of Kunming Medical University, Yunnan Cancer Hospital, Yunnan Cancer Center Kunming China

**Keywords:** colorectal cancer, perioperative, prognosis, skeletal muscle index

## Abstract

**Background:**

The association between perioperative changes in the skeletal muscle index (SMI) and colorectal cancer (CRC) outcomes remains unclear. We aim to explore perioperative change patterns of SMI and evaluate their effects on long‐term outcomes in CRC patients.

**Methods:**

This retrospective cohort study included Stage I–III CRC patients who underwent curative resection between 2012 and 2019. SMI at the third lumbar vertebra level was calculated using computed tomography scans. Optimal cut‐off values for SMI were defined separately for males and females and classified as high or low preoperatively and at 3, 6, 9 and 12 months postoperatively. SMI status was further categorized into different perioperative SMI change patterns: high_pre_–high_post_, high_pre_–low_post_, low_pre_–high_post_ and low_pre_–low_post_. The association with recurrence‐free survival (RFS) and overall survival (OS) was examined using Cox proportional hazards models.

**Results:**

A total of 2222 patients (median [interquartile range] age, 60.00 [51.00–68.00] years; 1302 (58.60%) men; 222 (9.99%) with preoperative low SMI) were evaluated. During a median follow‐up of 60 months, 375 patients (16.88%) died, and 617 patients (27.77%) experienced a recurrence. Multivariate Cox model analysis showed that, compared to patients with high_pre_–high_post_, those with high_pre_–low_post_ (HR = 3.32, 95% CI: 1.60–6.51; HR = 2.54, 95% CI: 1.03–6.26; HR = 2.93, 95% CI: 1.19–7.19, all *p* < 0.05) had significantly worse RFS and OS (HR = 4.07, 95% CI: 1.55–10.69; HR = 4.78, 95% CI: 1.40–16.29; HR = 9.69, 95% CI: 2.53–37.05, all *p* < 0.05), at postoperative 6, 9 and 12 months, respectively. Patients with low_pre_–low_post_ were an independent prognostic factor for worse OS at postoperative 12 months (HR = 3.20, 95% CI: 1.06–9.71, *p* = 0.040). Patients with low_pre_–high_post_ had similar risk of RFS compared to those with high_pre_–high_post_ at postoperative 3, 6 and 12 months (HR = 1.49, 95% CI: 0.75–2.98; HR = 1.05, 95% CI: 0.45–2.43; HR = 1.36, 95% CI: 0.31–6.06, all *p* > 0.05) and similar risk of OS at postoperative 3, 6, 9 and 12 months (all *p* > 0.05).

**Conclusions:**

Patients with a high preoperative SMI that decline postoperatively have poor RFS and OS. Consistently low SMI also correlates with worse OS. Patients with low SMI but increased after resection are not an indicator of better prognosis. Routine measurement of postoperative, rather than preoperative, SMI is warranted. Patients with low SMI are at an increased risk for recurrence and death, especially within the first year after surgery.

## 1 Introduction

Colorectal cancer (CRC) is the third leading cause of cancer‐related death globally [1]. The prognosis of CRC depends on several factors, including the TNM stage, location, molecular features and therapy responses [2]. However, these factors are not always sufficient to predict the prognosis or the optimal treatment strategy [3]. Therefore, new prognostic markers are urgently needed to better predict patients' survival and stratify them accordingly.

In CRC patients, body composition has been associated with postoperative complications and overall survival (OS) [4, 5]. Skeletal muscle index (SMI) is currently one of the most widely used and validated body composition parameters for prognosticating cancer outcomes [6]. SMI is calculated from computed tomography (CT) images by dividing the cross‐sectional area of skeletal muscle at the third lumbar vertebra (L3) by height squared [7]. Preoperative low SMI is an independent predictor for poor CRC prognosis [8, 9, 10]. Patients with preoperative low SMI could exhibit higher chemotherapy toxicity and poorer compliance and experience poor outcomes [11]. Besides, surgery could result in a significant loss of skeletal muscle [12, 13]. Perioperative skeletal muscle change may reflect the effect of surgery, inflammation, nutritional status, physical activity and adjuvant therapy on muscle mass and function and thus affect the outcomes of oncological patients. Though recent studies reported that postoperative SMI change was associated with CRC patients' outcomes [14, 15], there is lack of comprehensive understanding of the perioperative SMI change patterns of CRC patients. In addition, the association between SMI change patterns during the first year after surgery and long‐term CRC prognosis remains unknown.

In this study, we sought to explore perioperative SMI change patterns and investigate their association with recurrence‐free survival (RFS) and overall survival (OS) in CRC patients who underwent radical resection. To this end, we evaluated the prognostic value of SMI at five time points, including the preoperative baseline, postoperative 3, 6, 9 and 12 months. We also proposed four perioperative SMI change patterns, that is, preoperative high to postoperative high (abbreviated as high_pre_–high_post_), preoperative high to postoperative low (abbreviated as high_pre_–low_post_), preoperative low to postoperative high (abbreviated as low_pre_–high_post_) and preoperative low to postoperative low (abbreviated as low_pre_–low_post_) and investigated their association with long‐term CRC outcomes.

## 2 Methods

### 2.1 Patients and Study Design

**FIGURE 1:**
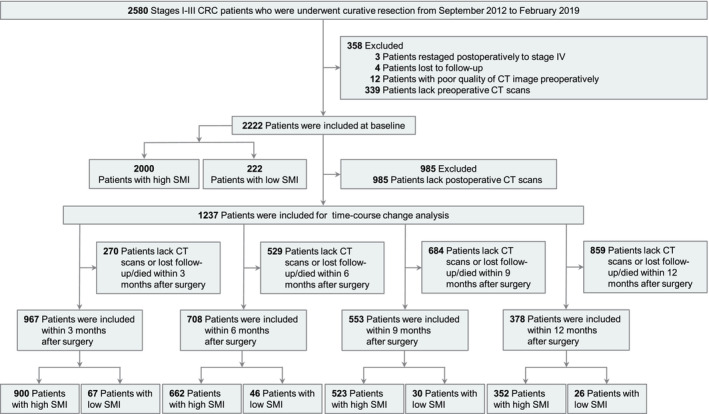
Patient selection flowchart.

The study protocol was approved by the ethics committee of Yunnan Cancer Hospital. The requirement for informed consent was waived by the ethics committee due to the retrospective nature of the study. All data were anonymized. The study included all consecutive stage I–III patients meeting the inclusion and exclusion criteria from September 2012 to February 2019 at Yunnan Cancer Hospital. Patients restaged postoperatively to stage IV, lost to follow‐up, poor quality of CT image preoperatively or lack preoperative CT scans were excluded. The detailed criteria for inclusion and exclusion were shown in Figure 1. This study followed the reporting guidelines of the Strengthening the Reporting of Observational Studies in Epidemiology [16].

### 2.2 Skeletal Muscle Assessment and CT Image Acquisition

CT cross‐sectional imaging is suggested as the preferred method to analyse muscle mass in cancer patients [17], and thus, CT scans were used to quantify skeletal muscle area in this study. CT cross‐sectional imaging was retrospectively collected from existing patient records. As the most pronounced loss of muscle mass occurs during the first year after surgery and remains stable after that [13, 18], the aim of this study was to observe changes in muscle mass during the first year postoperatively. CT scanning parameters can be found in Table S1.

Single‐slice muscle area at the third lumbar (L3) vertebra is strongly correlated with whole‐body volume of muscle tissue [19] and has been extensively used in oncology settings [17, 20]. The skeletal muscle area (cm^2^) at the L3 vertebra measured on cross‐sectional abdominal CT images was analysed using sliceOmatic software (Version 4.3; Tomovision, Montreal, Quebec, Canada) [4, 21]. The skeletal muscles at the L3 vertebra included the psoas, erector spinae, quadratus lumborum, transversus abdominis, external and internal oblique and rectus abdominis muscles. The regions of interest were manually adjusted to match the actual muscle boundaries by two abdominal radiologists blinded to the clinical data, and the skeletal muscle areas were automatically computed. When the correlation coefficient of the skeletal muscle area between the two manual measurements was greater than or equal to 0.90, the average was adopted as the finally result; otherwise, an additional measurement was conducted by a third radiologist (with > 25 years of clinical experience in CT scanning and image post‐processing) and was used as the final result. Subsequently, the skeletal muscle area was normalized by height in meter squared (m^2^) to calculate the SMI (cm^2^/m^2^).

### 2.3 Definition of SMI Group and SMI Change Patterns

Differences in skeletal muscle and adipose tissue distribution between male and female patients exist [22]. To determine our own cut‐off value for SMI, we used the same method from previous studies [6, 7, 23]. The cut‐off points for a continuous variable (in this case, L3 SMI) from the sex‐specific strata were selected using the X‐Tile software (Version 3.6.1) [7, 24]. For each candidate cut‐off point, we compared the difference in RFS between the high and low SMI groups and calculated the log‐rank statistic. Only the cut‐off point with the largest absolute value of the log‐rank statistic was selected to define the different SMI groups [25].

Then we used the cut‐off point to define the patients as with low or high SMI both preoperatively and postoperatively 3, 6, 9 and 12 months and divided them into four groups according to their SMI change. Throughout the text, these patterns were defined: high_pre_–high_post_, high_pre_–low_post_, low_pre_–high_post_ and low_pre_‐low_post_.

### 2.4 Covariates

Covariates were retrospectively obtained from the electronic medical record after obtaining explicit patient consent or following appropriate privacy safeguards, including age, sex, BMI, smoking history, drinking history, hypertension, diabetes, coronary heart disease, chronic obstructive pulmonary disease (COPD), Eastern Cooperative Oncology Group (ECOG) grade, weight change at postoperative 3 months, preoperative serum albumin, primary site (colon or rectum), pathological stage (I–III), tumour differentiation, histologic type (mucinous type or non‐mucinous type), T stage, N stage, lymph node yield (< 12 or ≥ 12), lymph vascular invasion status (yes or no), perineural invasion (yes or no), tumour deposit (yes or no) and adjuvant chemotherapy (yes or no).

### 2.5 Follow‐Up and Outcome

A follow‐up protocol was established following the Chinese Society of Clinical Oncology Guidelines for the Diagnosis and Treatment of CRC. Patients were followed up regularly either by phone or in person at the hospital prospectively. Serum carcinoembryonic antigen (CEA) was examined at 3‐ to 6‐month intervals during the first 2 years after surgery and every 6 months thereafter. In addition, contrast‐enhanced CT of the chest, abdomen and pelvis was performed at a minimum of every 6 months for at least 3 years. Colonoscopy was performed 1 year after surgery and every 2–5 years thereafter unless warranted otherwise (e.g., identification of advanced adenomas). The collected follow‐up information included the adjuvant chemotherapy therapy status, tumour recurrence, time of recurrence, patient survival time and time of death. All follow‐up information obtained through telephone interviews adhered to the principles of patient privacy protection.

The primary outcome was RFS. Recurrence included local recurrence and distant metastases, which were confirmed by biopsy tests, imaging examinations or histological analysis. RFS was calculated from the date of surgery until the date of cancer recurrence, death or last follow‐up visit. The secondary outcome was OS. Data from patients who were lost to follow‐up were censored at the date of last known contact.

### 2.6 Statistical Analysis

We assessed for missing values before data analysis. The missing data were excluded from the analysis to ensure that only eligible data were included. We presented data as mean ± standard deviation (SD) for those following a normal distribution and continuous variables as median with interquartile range (IQR) for non‐normally distributed data. We applied independent samples *t*‐tests for comparing means of normally distributed variables between two independent groups and the Wilcoxon rank‐sum tests for comparing medians of non‐normally distributed data between two independent groups. When paired data were analysed, paired *t*‐tests (for normally distributed differences) or Wilcoxon signed‐rank tests (for non‐normally distributed differences) were employed. Categorical variables were expressed as totals and percentages and compared using the chi‐square test or Fisher's exact test.

The Kaplan–Meier RFS and OS curves were compared using the log‐rank test. Univariate and multivariate Cox proportional risk regression models were used to assess the associations between the SMI status at a solitary time point or SMI change patterns and survival after confirming the proportional hazard assumption. Two multivariate analyses models were used. Model 1 was adjusted for sex, age (continuous), smoking history, drinking history, hypertension, diabetes, coronary heart disease, COPD and ECOG grade. Model 2 was adjusted for Model 1 plus weight change at postoperative 3 months, preoperative serum albumin, primary site, pathological stage, lymph node yield, tumour differentiation, histologic type, perineural invasion, lymph vascular invasion, tumour deposit and adjuvant chemotherapy. The hazard ratio (HR) and 95% confidence intervals (CIs) were calculated and determined. A *p* value below 0.05 was considered statistically significant. The data were analysed preoperatively, at postoperative 3, 6, 9 and 12 months, respectively. Data processing and analysis were performed using R software (Version 4.2.1).

## 3 Results

### 3.1 Baseline Clinical and Pathological Characteristics

A total of 2580 consecutive patients (1493 [57.87%] male; median [IQR] age, 60 [51–67] years) who underwent curative resection for stage I to III CRC were identified. A flowchart of the study was shown in Figure 1. Patients were excluded if they had restaged postoperatively to stage IV (*n* = 3), lost to follow‐up (*n* = 4), with poor quality of CT image preoperatively (*n* = 12) and lack preoperative CT scans (*n* = 339). A comparison of the baseline characteristics between the excluded and included analysis populations was shown in Table S2.

A total of 2222 patients were included in the study, including 1302 (58.60%) males, with mean age of 58.93 ± 11.70 years and long‐term follow‐up (median intervals: 60 months, [IQR]: 58–61.6 months). During the follow‐up period, 375 patients (16.88%) died and were identified with an incidence density of 36.54 per 1000 person‐years, and 617 patients (27.77%) experienced a recurrence with an incidence density of 66.75 per 1000 person‐years. The cut‐off values obtained for this study were as follows: ≥ 39.4 cm^2^/m^2^ (high SMI) and < 39.4 cm^2^/m^2^ (low SMI) for male and ≥ 30.5 cm^2^/m^2^ (high SMI) and < 30.5 cm^2^/m^2^ (low SMI) for female. Two hundred twenty‐two (9.99%) patients with preoperative low SMI were significantly older, had lower BMI and had more COPD than those with preoperative high SMI (all *p* < 0.05; Table 1). Of the 1237 (55.67%) patients with postoperative CT scan, 967 (43.53%), 708 (31.86%), 553 (24.89%) and 378 (17.01%) patients were included in postoperative 3, 6, 9 and 12 months, respectively. The demographic and clinicopathological baseline characteristics of the populations with and without postoperative CT scan data are presented in Table S3. Patients who underwent postoperative CT scans tended to be younger and exhibited a higher BMI (all *p* < 0.05).

**TABLE 1:** Demographic and clinicopathological baseline characteristics of included patients.

Parameter	All patients^a^ (*n* = 2222)	Skeletal muscle index		*p* ^b^
		High group^a^ (*n* = 2000)	Low group^a^ (*n* = 222)	
Age, years				
Median (IQR)	60.00 (51.00, 68.00)	59.00 (51.00, 67.00)	68.00 (59.25, 74.00)	< 0.001
Sex, *n* (%)				0.075
Female	920 (41.40)	841 (42.05)	79 (35.59)	
Male	1302 (58.60)	1159 (57.95)	143 (64.41)	
BMI (kg/m^b^)				
Median (IQR)	22.59 (20.76, 24.92)	22.97 (20.82, 25.22)	20.45 (18.30, 21.08)	< 0.001
Smoking history, *n* (%)				0.982
Yes	556 (25.02)	500 (25.00)	56 (25.23)	
No	1644 (73.99)	1480 (74.00)	164 (73.87)	
Unknown	22 (0.99)	20 (1.00)	2 (0.90)	
Drinking history, *n* (%)				0.169
Yes	419 (18.86)	370 (18.50)	49 (22.07)	
No	1724 (77.59)	1555 (77.75)	169 (76.13)	
Unknown	79 (3.56)	75 (3.75)	4 (1.80)	
Hypertension, *n* (%)				0.343
Yes	535 (24.08)	475 (23.75)	60 (27.03)	
No	1680 (75.61)	1519 (75.95)	161 (72.52)	
Unknown	7 (0.32)	6 (0.30)	1 (0.45)	
Diabetes, *n* (%)				0.924
Yes	188 (8.46)	171 (8.55)	17 (7.66)	
No	2022 (91.00)	1818 (90.90)	204 (91.89)	
Unknown	12 (0.54)	11 (0.55)	1 (0.45)	
Coronary heart disease, *n* (%)				0.652
Yes	65 (2.93)	57 (2.85)	8 (3.60)	
No	2147 (96.62)	1934 (96.70)	213 (95.95)	
Unknown	10 (0.45)	9 (0.45)	1 (0.45)	
COPD, *n* (%)				< 0.001
Yes	21 (0.95)	14 (0.70)	7 (3.15)	
No	2182 (98.20)	1972 (98.60)	210 (94.59)	
Unknown	19 (0.86)	14 (0.70)	5 (2.25)	
ECOG grade, *n* (%)				0.441
0	1160 (52.21)	1047 (52.35)	113 (50.90)	
1	961 (43.25)	865 (43.25)	96 (43.24)	
2	61 (2.75)	55 (2.75)	6 (2.70)	
≥ 3	40 (1.80)	33 (1.65)	7 (3.15)	
Weight change at postoperative 3 months (kg)				
Median (IQR)	−3.00 (−5.00, −1.00)	−3.00 (−5.00, −1.00)	−2.50 (−5.00, 0.00)	0.194
Weight change at postoperative 3 months, *n* (%)				0.031
Decline	1227 (55.22)	1122 (56.10)	105 (47.30)	
Increase	381 (17.15)	340 (17.00)	41 (18.47)	
Unknown	614 (27.63)	538 (26.90)	76 (34.23)	
Preoperative serum albumin (g/L)				
Median (IQR)	45.00 (42.00, 47.00)	45.00 (42.00, 47.00)	43.00 (39.00, 46.00)	< 0.001
Preoperative serum albumin group, *n* (%)				< 0.001
< 35	86 (3.87)	65 (3.25)	21 (9.46)	
≥ 35	2132 (95.95)	1931 (96.55)	201 (90.54)	
Unknown	4 (0.18)	4 (0.20)	0 (0.00)	
Primary site, *n* (%)				0.336
Colon	1058 (47.61)	945 (47.25)	113 (50.90)	
Rectum	1164 (52.39)	1055 (52.75)	109 (49.10)	
Pathological stage, *n* (%)				0.485
I	528 (23.76)	481 (24.05)	47 (21.17)	
II	847 (38.12)	755 (37.75)	92 (41.44)	
III	847 (38.12)	764 (38.20)	83 (37.39)	
Tumour differentiation, *n* (%)				0.737
Well + moderate	603 (27.14)	547 (27.35)	56 (25.23)	
Poor	1429 (64.31)	1284 (64.20)	145 (65.32)	
Unknown	190 (8.55)	169 (8.45)	21 (9.46)	
Histologic type, *n* (%)				0.197
Mucinous type	2090 (94.06)	1886 (94.30)	204 (91.89)	
Non‐mucinous type	132 (5.94)	114 (5.70)	18 (8.11)	
T stage, *n* (%)				0.287
T1	186 (8.37)	173 (8.65)	13 (5.86)	
T2	424 (19.08)	381 (19.05)	43 (19.37)	
T3	1513 (68.09)	1361 (68.05)	152 (68.47)	
T4	99 (4.46)	85 (4.25)	14 (6.31)	
N stage, *n* (%)				0.252
N0	1358 (61.12)	1220 (61.00)	138 (62.16)	
N1	623 (28.04)	556 (27.80)	67 (30.18)	
N2	241 (10.85)	224 (11.20)	17 (7.66)	
Lymph node yield, *n* (%)				0.088
< 12	458 (20.61)	402 (20.10)	56 (25.23)	
≥ 12	1764 (79.39)	1598 (79.90)	166 (74.77)	
Lymph vascular invasion, *n* (%)				0.423
Yes	174 (7.83)	161 (8.05)	13 (5.86)	
No	351 (15.80)	312 (15.60)	39 (17.57)	
Unknown	1697 (76.37)	1527 (76.35)	170 (76.58)	
Perineural invasion, *n* (%)				0.800
Yes	54 (2.43)	50 (2.50)	4 (1.80)	
No	447 (20.12)	401 (20.05)	46 (20.72)	
Unknown	1721 (77.45)	1549 (77.45)	172 (77.48)	
Tumour deposit, *n* (%)				1.000
Yes	204 (9.18)	184 (9.20)	20 (9.01)	
No	2018 (90.82)	1816 (90.80)	202 (90.99)	
Adjuvant chemotherapy, *n* (%)				0.185
Yes	1259 (56.66)	1143 (57.15)	116 (52.25)	
No	963 (43.34)	857 (42.85)	106 (47.75)	

Abbreviations: IQR, interquartile range; SD, standard deviation.

^a^ *n* (%); median (IQR).

^b^ Pearson's chi‐squared test; Wilcoxon rank‐sum test; Fisher's exact test.

In our study, baseline CT scans (*n* = 2222) were performed on average 8.94 days before surgery (median [IQR]: 8 [6, 12]). Postoperative CT scans on average 3.09 months at postoperative 3 months (*n* = 1425) (median [IQR]: 3.13 [2.47–3.73]), 5.85 months at postoperative 6 months (*n* = 900) (median [IQR]: 5.83 [5.13–6.47]), 9.11 months at postoperative 9 months (*n* = 588) (median [IQR]: 9.27 [8.43–9.80]) and 11.66 months at postoperative 12 months (*n* = 382) (median [IQR]: 11.16 [11.10–12.13]) were performed.

### 3.2 Perioperative Changes in SMI

**FIGURE 2:**
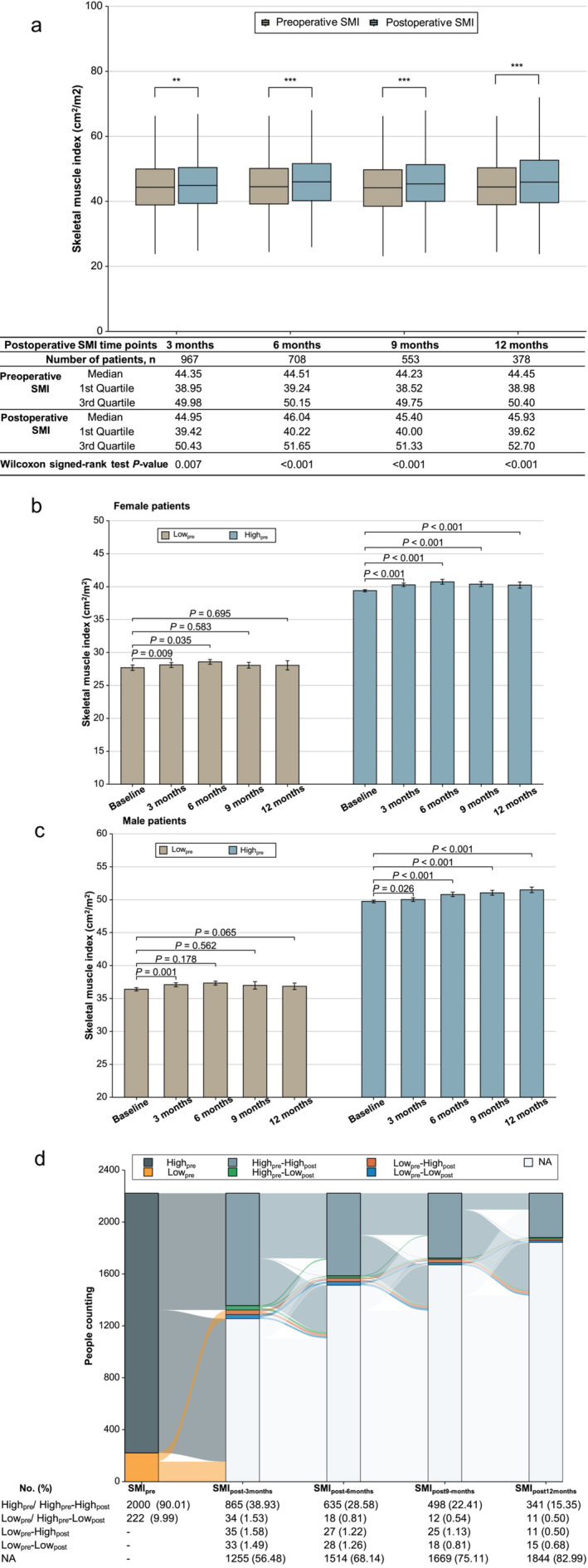
Time‐course changes in the average skeletal muscle index (SMI) in patients before and after undergoing curative resection. Bars represent the mean SMI values, and error bars represent the standard error of the mean (SEM). (a) Box‐and‐whisker plots show changes in patients' SMI before and after surgery, and statistical significance was calculated by the Wilcoxon signed‐rank tests. (b) Female patients with and without low SMI time changes of SMI, and statistical significance was calculated by the Wilcoxon rank‐sum tests. (c) Male patients with and without low SMI time changes of skeletal muscle index (SMI), and statistical significance was calculated by the Wilcoxon rank‐sum tests. (d) A Sankey diagram showed the longitudinal changes and number (%) in SMI for patients grouped by different change patterns. The percentages are based on the number of patients with available preoperative CT scans (*n* = 2222).

The time‐course changes in the average SMI in patients before and after surgery was shown in Figure 2. Figure 2a illustrated that the preoperative average SMI increased in all patients at 3, 6, 9 and 12 months postoperatively, respectively (all *p* < 0.05). For female patients with preoperative low SMI, the average SMI increased from 27.68 cm^2^/m^2^ preoperatively to 28.11, 28.57, 28.06 and 28.05 cm^2^/m^2^ at 3, 6, 9 and 12 months postoperatively, respectively. In female patients with preoperative high SMI, the average SMI increased from 39.38 cm^2^/m^2^ preoperatively to 40.28, 40.74, 40.39 and 40.24 cm^2^/m^2^ at the same time points (Figure 2b). For male patients with preoperative low SMI, the average SMI increased from 36.40 cm^2^/m^2^ preoperatively to 37.09, 37.34, 37.00 and 36.85 cm^2^/m^2^ at 3, 6, 9 and 12 months postoperatively, respectively. In male patients with preoperative high SMI, the average SMI increased from 49.73 cm^2^/m^2^ preoperatively to 50.03, 50.81, 51.05 and 51.48 cm^2^/m^2^ at the same time points (Figure 2c).

In the preoperative low SMI group, males showed a significant increase in SMI only at 3 months postoperatively (*p* < 0.05; Figure 2c), whereas females showed significant increases at both 3 and 6 months (all *p* < 0.05; Figure 2b). However, no statistically significant increase was observed at 12 months for either sex (all *p* > 0.05; Figure 2b,c). In the preoperative high SMI group, both males and females exhibited significant increases in SMI at 3–12 months postoperatively compared to preoperative SMI (all *p* < 0.05; Figure 2b,c).

Figure 2d illustrates the number (%) of patients' SMI changes at the five time points. Among 222 patients with preoperative low SMI, 35, 27, 25 and 11 were changed to high SMI, and 33, 28, 18 and 15 remained with low SMI at postoperative 3, 6, 9 and 12 months, respectively. Among 2000 patients with preoperative high SMI, 34, 18, 12 and 11 transitioned to low SMI, whereas 865, 635, 498 and 341 maintained high SMI, at the same postoperative points.

Tables S4–S7 detail the clinical characteristics of patients exhibiting different SMI change patterns at postoperative 3, 6, 9 and 12 months, respectively. Patients categorized within the low_pre_–low_post_ group are consistently older and exhibit a lower BMI across all assessed postoperative periods. Additionally, a significant difference in T stage has been observed between the four SMI change patterns groups at both 3 and 9 months postoperatively (all *p* < 0.05). Figure S1 showed representative axial CT images of patients with different SMI change patterns.

### 3.3 SMI Was an Independent Prognostic Factor at Different Perioperative Time Points for CRC Outcomes

Patient with low SMI had a significantly lower RFS compared to patients with high SMI at postoperative 6, 9 and 12 months (all log‐rank *p* < 0.05; Figure S2a–d). Specifically, the 5‐year RFS rate for the 46 patients with low SMI was 62.03% (95% CI, 49.27%–78.09%) compared with 72.84% (95% CI, 69.23%–76.62%) for the 662 patients with high SMI in postoperative 6 months (Figure S2b). At postoperative 9 months, the 5‐year RFS rate for the 30 patients with low SMI was 63.33% (95% CI, 48.24%–83.15%) compared with 75.29% (95% CI, 71.51%–79.27%) for the 523 patients with high SMI (Figure S2c). At postoperative 12 months, the 5‐year RFS rate for the 26 patients with low SMI was 53.85% (95% CI, 37.72%–76.86%) compared with 76.22% (95% CI, 71.48%–81.28%) for the 352 patients with high SMI (Figure S2d).

Figure S3a–d also showed that low SMI patients had a lower OS compared to patients with high SMI at postoperative 6, 9 and 12 months (all log‐rank *p* < 0.05). Specifically, the 5‐year OS rates for patients with low SMI were 72.13% (95% CI, 56.69%–91.77%) at 6 months, 79.86% (95% CI, 66.67%–95.66%) at 9 months and 57.12% (95% CI, 36.58%–89.17%) at 12 months, compared to 86.76% (95% CI, 83.80%–89.82%), 88.85% (95% CI, 85.83%–91.97%) and 88.04% (95% CI, 83.84%–92.45%) for patients with high SMI, respectively.

Multivariable analysis showed low SMI was an independent risk factor for poor RFS at preoperative baseline, postoperative 6, 9 and 12 months (all *p* < 0.05; Table S8), as well as OS, at postoperative 6 and 12 months (Table S9).

### 3.4 SMI Change Pattern Was Also an Independent Prognostic Factor for CRC Outcomes

**FIGURE 3:**
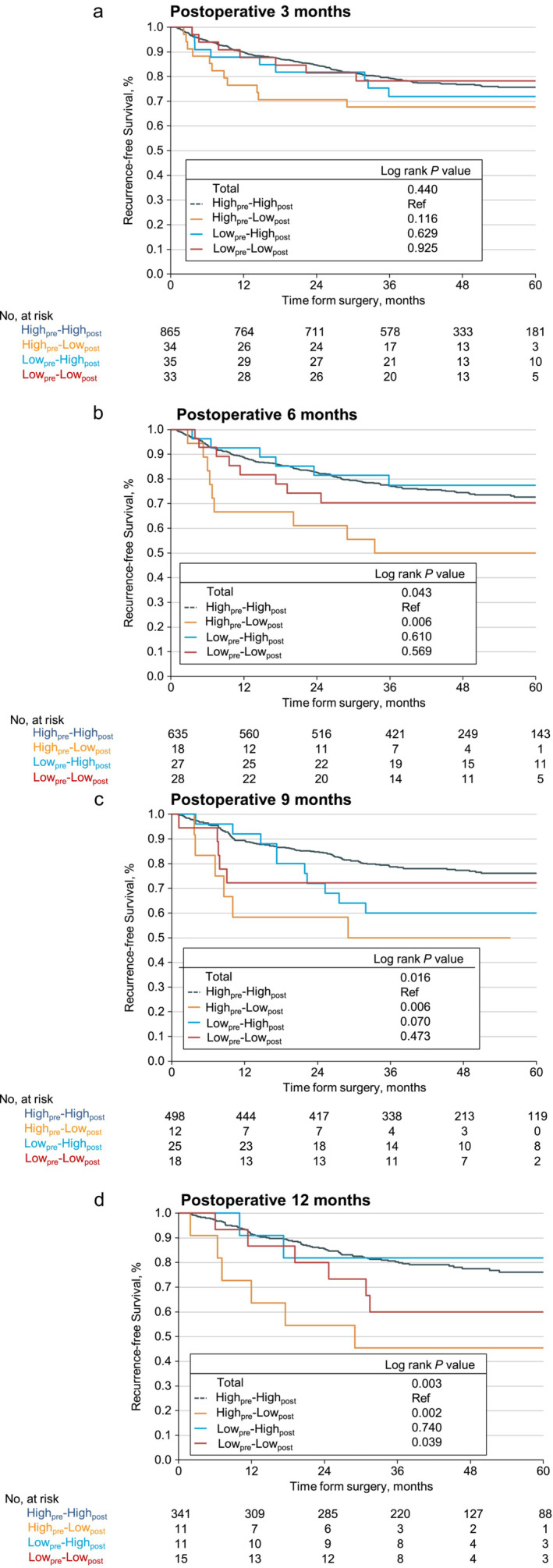
Kaplan–Meier curves for recurrence‐free survival (RFS) of the four skeletal muscle index (SMI) change patterns. Statistical significance was calculated by the log‐rank test: (a) at postoperative 3 months, (b) at postoperative 6 months, (c) at postoperative 9 months and (d) at postoperative 12 months.

We further analysed patient survival according to the SMI change patterns at four postoperative time points. Figure 3a–d presented the RFS of four groups at different postoperative time points.

Compared to those with high_pre_–high_post_, patients with high_pre_–low_post_ had lower RFS at postoperative 6 months (3‐year RFS 77.16% [95% CI, 73.93%–80.52%] vs. 50.00% [95% CI, 31.50%–79.36%], log‐rank *p* = 0.006; Figure 3b), as well as postoperative 9 month (3‐year RFS 78.66% [95% CI, 75.12%–82.37%] vs. 50.00% [95% CI, 28.40%–88.04%], log‐rank *p* = 0.006; Figure 3c) and 12 months (3‐year RFS 80.23% [95% CI, 76.06%–84.63%] vs. 45.45% [95% CI, 23.79%–86.84%], log‐rank *p* = 0.002; Figure 3d). Patients with low_pre_–low_post_ also had lower RFS at postoperative 12 months compared to high_pre_–high_post_ group (5‐year RFS 76.03% [95% CI, 71.19%–81.20%] vs. 60.00% [95% CI, 36.69%–90.70%], log‐rank *p* = 0.039; Figure 3d).

**FIGURE 4:**
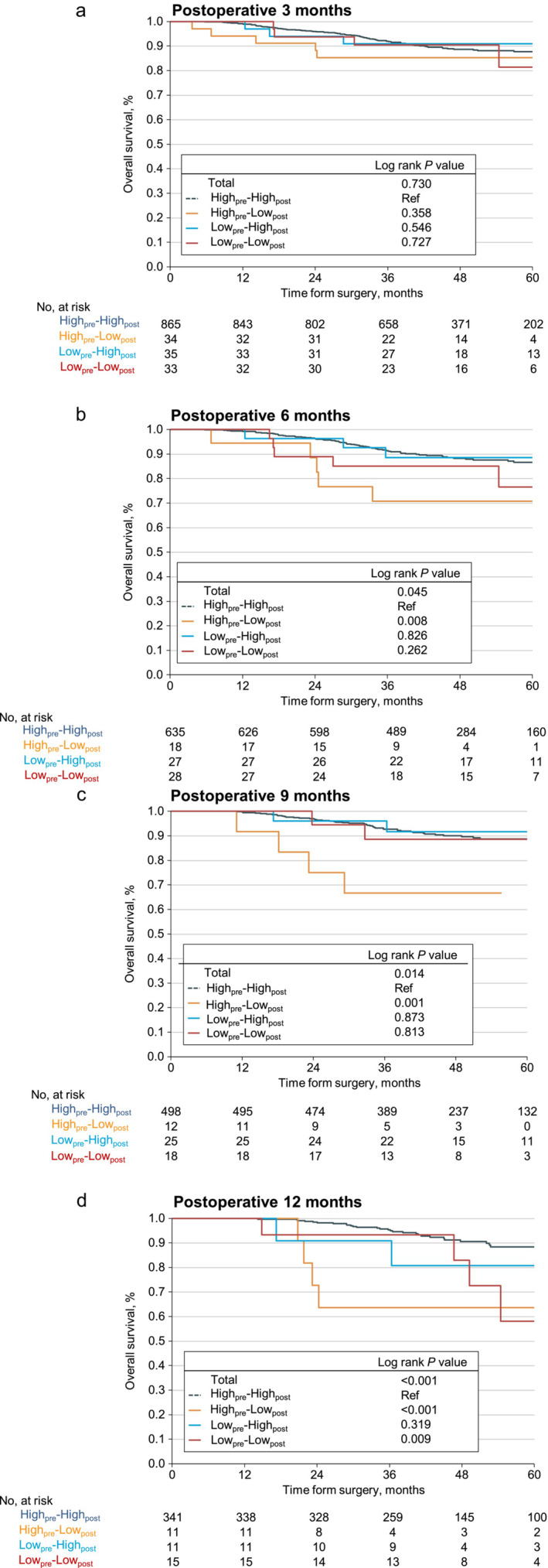
Kaplan–Meier curves for overall survival of the four skeletal muscle index (SMI) change patterns. Statistical significance was calculated by the log‐rank test: (a) at postoperative 3 months, (b) at postoperative 6 months, (c) at postoperative 9 months and (d) at postoperative 12 months.

Figure 4a–d presented the OS of four groups at different postoperative time points. Compared to those with high_pre_–high_post_, patients with high_pre_–low_post_ had lower OS at postoperative 6, 9 and 12 months, and so did patients with low_pre_–low_post_ at postoperative 12 months (all log‐rank *p* < 0.05). The high_pre_–low_pos_ group 3‐year OS compared to the high_pre_–high_post_ group at 6 months (70.83% [95% CI, 52.25%–96.02%] vs. 91.31% [95% CI, 89.09%–93.58%]; Figure 4b), 9 months (66.67% [95% CI, 44.68%–99.46%] vs. 92.65% [95% CI, 90.33%–95.03%]; Figure 4c) and 12 months (95.33% [95% CI, 93.04%–97.67%] vs. 63.64% [95% CI, 40.71%–99.47%] Figure 4d). The low_pre_–low_post_ group also had lower OS at 12 months (5‐year OS 58.07% [95% CI, 32.63%–100%] vs. 88.30% [95% CI, 84.04%–92.78%], log‐rank *p* = 0.009; Figure 4d).

Figure S4a shows the HRs of univariate analysis for RFS and OS increased in both the high_pre_–low_post_ and low_pre_–low_post_ groups. Specifically, the HRs for RFS, where the high_pre_–low_post_ group had HRs of 1.62 (95% CI, 0.88–2.97) at 3 months, 2.48 (95% CI, 1.27–4.86) at 6 months, 2.96 (95% CI, 1.30–6.73) at 9 months and 3.55 (95% CI, 1.54–8.16) at 12 months compared to the high_pre_–high_post_ group. Similarly, high_pre_–low_post_ group showed an increased risk of death over time, with HRs of 1.54 (95% CI, 0.62–3.78) at 3 months, 3.18 (95% CI, 1.28–7.89) at 6 months, 4.70 (95% CI, 1.69–13.05) at 9 months and 6.14 (95% CI, 2.16–17.5) at 12 months (Figure S4b). The HRs of each SMI change patterns group also indicated that the risk of recurrence and death was highest in the high_pre_–low_post_ group within the first year postoperatively (Figure S4).

Multivariate Cox regression model analysis confirmed that SMI change pattern was also an independent risk factor for CRC outcomes. Compared to patients with high_pre_–high_post_, patients with high_pre_–low_post_ had worse RFS, as well as worse OS, at postoperative 6, 9 and 12 months, respectively (all *p* < 0.05; Tables 2 and 3). And patients with low_pre_–low_post_ had worse OS at postoperative 12 months (*p* = 0.040; Table 3). But patients with low_pre_–high_post_ had the similar risk of RFS and OS compared to those with high_pre_–high_post_ at postoperative 3, 6 and 12 months (all *p* > 0.05; Tables 2 and 3).

**TABLE 2:** Univariate and multivariate analysis of changes in skeletal muscle index and recurrence‐free survival at postoperative 3, 6, 9 and 12 months.

Variable	Univariate analysis			Multivariate analysis (M1)^a^		Multivariate analysis (M2)^b^	
	No. (%)	HR (95%CI)	*p*	HR (95%CI)	*p*	HR (95%CI)	*p*
Postoperative 3 months							
High_pre_–High_post_	865 (89.45)	Ref		Ref		Ref	
High_pre_–Low_post_	34 (3.52)	1.62 (0.88, 2.97)	0.119	1.58 (0.86, 2.93)	0.143	1.63 (0.87, 3.03)	0.125
Low_pre_–High_post_	35 (3.62)	1.18 (0.60, 2.30)	0.629	1.30 (0.66, 2.57)	0.442	1.49 (0.75, 2.98)	0.255
Low_pre_–Low_post_	33 (3.41)	0.96 (0.45, 2.04)	0.920	1.03 (0.48, 2.20)	0.948	1.15 (0.53, 2.48)	0.721
Postoperative 6 months							
High_pre_–High_post_	635 (89.69)	Ref		Ref		Ref	
High_pre_–Low_post_	18 (2.54)	2.48 (1.27, 4.86)	0.008	2.72 (1.36, 5.43)	0.005	3.23 (1.6, 6.51)	0.001
Low_pre_–High_post_	27 (3.81)	0.81 (0.36, 1.83)	0.616	0.88 (0.38, 1.99)	0.750	1.05 (0.45, 2.43)	0.914
Low_pre_–Low_post_	28 (3.95)	1.23 (0.60, 2.49)	0.573	1.17 (0.56, 2.44)	0.679	1.45 (0.66, 3.15)	0.353
Postoperative 9 months							
High_pre_–High_post_	498 (90.05)	Ref		Ref		Ref	
High_pre_–Low_post_	12 (2.17)	2.96 (1.30, 6.73)	0.010	3.04 (1.28, 7.23)	0.012	2.54 (1.03, 6.26)	0.043
Low_pre_–High_post_	25 (4.52)	1.80 (0.94, 3.43)	0.076	2.06 (1.06, 4.00)	0.033	2.09 (1.06, 4.14)	0.034
Low_pre_–Low_post_	18 (3.25)	1.39 (0.57, 3.39)	0.476	1.37 (0.55, 3.42)	0.495	1.85 (0.73, 4.70)	0.192
Postoperative 12 months							
High_pre_–High_post_	341 (90.21)	Ref		Ref		Ref	
High_pre_–Low_post_	11 (2.91)	3.55 (1.54, 8.16)	0.003	3.36 (1.42, 7.96)	0.006	2.93 (1.19, 7.19)	0.019
Low_pre_–High_post_	11 (2.91)	0.79 (0.19, 3.20)	0.738	0.91 (0.21, 3.89)	0.901	1.36 (0.31, 6.06)	0.687
Low_pre_–Low_post_	15 (3.97)	2.20 (1.02, 4.78)	0.045	2.06 (0.81, 5.22)	0.130	1.95 (0.76, 5.01)	0.163

Abbreviations: CI, confidence interval; HR, hazard ratio; Ref, reference.

^a^ Multivariate analysis (M1) was adjusted for sex, age (continuous), smoking history, drinking history, hypertension, diabetes, coronary heart disease, COPD and ECOG grade.

^b^ Multivariate analysis (M2) was adjusted for multivariate analysis (M1) plus weight change at postoperative 3 months, preoperative serum albumin (binary), primary site, pathological stage, lymph node yield, tumour differentiation, histologic type, perineural invasion, lymph vascular invasion, tumour deposit and adjuvant chemotherapy.

**TABLE 3:** Univariate and multivariate analysis of changes in skeletal muscle index and overall survival at postoperative 3, 6, 9 and 12 months.

Variable	Univariate analysis			Multivariate analysis (M1)^a^		Multivariate analysis (M2)^b^	
	No. (%)	HR (95%CI)	*p*	HR (95%CI)	*p*	HR (95%CI)	*p*
Postoperative 3 months							
High_pre_–High_post_	865 (89.45)	Ref		Ref		Ref	
High_pre_–Low_post_	34 (3.52)	1.54 (0.62, 3.78)	0.349	1.53 (0.61, 3.83)	0.366	1.74 (0.69, 4.42)	0.242
Low_pre_–High_post_	35 (3.62)	1.32 (0.54, 3.24)	0.548	1.35 (0.54, 3.40)	0.520	1.48 (0.58, 3.78)	0.411
Low_pre_–Low_post_	33 (3.41)	1.20 (0.44, 3.26)	0.722	1.12 (0.41, 3.10)	0.820	1.36 (0.49, 3.81)	0.559
Postoperative 6 months							
High_pre_–High_post_	635 (89.69)	Ref		Ref		Ref	
High_pre_–Low_post_	18 (2.54)	3.18 (1.28, 7.89)	0.013	3.05 (1.19, 7.80)	0.020	4.07 (1.55, 10.69)	0.004
Low_pre_–High_post_	27 (3.81)	0.88 (0.28, 2.79)	0.825	0.88 (0.27, 2.83)	0.831	0.82 (0.23, 2.89)	0.752
Low_pre_–Low_post_	28 (3.95)	1.67 (0.68, 4.14)	0.266	1.60 (0.63, 4.08)	0.320	2.10 (0.79, 5.59)	0.139
Postoperative 9 months							
High_pre_–High_post_	498 (90.05)	Ref		Ref		Ref	
High_pre_–Low_post_	12 (2.17)	4.70 (1.69, 13.05)	0.003	4.96 (1.64, 14.97)	0.005	4.78 (1.40, 16.29)	0.012
Low_pre_–High_post_	25 (4.52)	1.11 (0.34, 3.55)	0.865	1.30 (0.39, 4.28)	0.669	1.27 (0.37, 4.34)	0.706
Low_pre_–Low_post_	18 (3.25)	1.19 (0.29, 4.89)	0.811	1.11 (0.27, 4.65)	0.885	0.87 (0.20, 3.83)	0.854
Postoperative 12 months							
High_pr_–High_post_	341 (90.21)	Ref		Ref		Ref	
High_pre_–Low_post_	11 (2.91)	6.14 (2.16, 17.5)	< 0.001	7.70 (2.45, 24.25)	< 0.001	9.69 (2.53, 37.05)	< 0.001
Low_pre_–High_post_	11 (2.91)	2.02 (0.48, 8.46)	0.334	1.94 (0.43, 8.81)	0.391	4.39 (0.85, 22.79)	0.078
Low_pre_–Low_post_	15 (3.97)	3.34 (1.30, 8.61)	0.012	3.76 (1.36, 10.36)	0.011	3.20 (1.06, 9.71)	0.040

Abbreviations: CI, confidence interval; HR, hazard ratio; Ref, reference.

^a^ Multivariate analysis (M1) was adjusted for sex, age (continuous), smoking history, drinking history, hypertension, diabetes, coronary heart disease, COPD and ECOG grade.

^b^ Multivariate analysis (M2) was adjusted for multivariate analysis (M1) plus weight change at postoperative 3 months, preoperative serum albumin (binary), primary site, pathological stage, lymph node yield, tumour differentiation, histologic type, perineural invasion, lymph vascular invasion, tumour deposit and adjuvant chemotherapy.

## 4 Discussion

In this study, we investigated the perioperative SMI change patterns of CRC patients and their association with long‐term survival outcomes. Our findings demonstrate that perioperative SMI change, as well as SMI status, is an independent prognostic factor in CRC patients. We found that low SMI at preoperative baseline, postoperative 6, 9 and 12 months, was an independent negative factor for RFS and OS in CRC patients. We also identified the dynamic change of SMI as another independent predictor for CRC prognosis. Specifically, SMI developed low postoperatively is associated with the poor RFS and OS. Patients with persistently low SMI are also significantly associated with poor OS. Conversely, patients with low preoperative SMI but elevated postoperative SMI had a similar prognosis, compared to patients with high preoperative and postoperative SMI. Patients developed low SMI postoperative are at increased risk for recurrence and death, especially within the first 12 months after surgery. Our study expands the existing evidence on perioperative SMI change and CRC outcomes by highlighting the additive effect of SMI change patterns, independent of preoperative SMI.

Current literature on the relationship between SMI and CRC prognosis primarily focuses on single preoperative or postoperative time points [8, 9, 10]. Studies examining the dynamic changes in SMI over time remain limited. We found that the dynamic change of SMI was correlated with CRC prognosis as shown by Lee et al. and Brown et al. [14, 15]. However, our study differed from Lee et al. in that we detailed the dynamic SMI changes at postoperative 1 year rather than 3 years and their effect on RFS and OS, and we proposed four SMI change patterns instead of two [14]. We categorized patients into four groups based on perioperative SMI changes: high_pre_–high_post_ (high SMI both before and after surgery), high_pre_–low_post_ (high SMI before surgery transitioning to low SMI after), low_pre_–high_post_ (low SMI before surgery improving to high SMI afterwards) and low_pre_–low_post_ (low SMI both before and after surgery). Brown et al. focused on muscle mass (muscle cross‐sectional area) and radiodensity, which were quantified using CT images obtained at diagnosis and after approximately 14 months [15]. The primary study outcome was all‐cause mortality. In contrast, our study emphasizes the change of SMI in the first postoperative year and its implications for RFS and OS, offering a novel perspective on the critical window for intervention to preserve muscle mass and improve patient outcomes.

Postoperative decline in SMI is a significant concern for surgical patients [14]. Our analysis revealed that patients with a high preoperative SMI but low postoperative SMI had a significantly higher risk of poor RFS and OS. Surgical trauma and inflammation seems to play a key role in the catabolic response, resulting in accelerated postoperative muscle loss [26, 27, 28, 29, 30]. Postoperative dietary restrictions and reduced appetite lead to insufficient protein and calorie intake, worsening muscle loss [30]. Physical inactivity during recovery exacerbates muscle atrophy, although metabolic changes, such as insulin resistance, impair muscle protein synthesis [27, 31]. Cancer‐related cachexia also significantly contributes to muscle wasting [32, 33]. The surgical removal of cancerous tissue, although essential for treatment, induced an inflammatory response that exacerbated muscle loss. Persistent postoperative inflammation, dietary insufficiencies and physical inactivity were linked to the decline in SMI. These factors also compromised immune function, impaired recovery and reduced the effectiveness of adjuvant treatments. Moreover, low SMI is associated with decreased quality of life [34], which would additionally lead to poor oncologic outcomes. This reduction in muscle mass can trigger a cycle of declining health, emphasizing the need for integrated postoperative care. Given these insights, it is critical to implement comprehensive postoperative strategies that include nutritional support, physical rehabilitation and monitoring for inflammatory responses. Interestingly, the patients with low SMI developed postoperatively exhibited a worse prognosis compared to patients with low SMI during the whole perioperative period. One possible explanation could be individuals with low SMI might have adapted to lower muscle mass over time with their bodies having developed compensatory mechanisms to maintain functionality [35]. In contrast, individuals transitioning from high SMI to low SMI are experiencing a recent loss of muscle mass, affecting their overall health and functionality to a greater extent [35]. Importantly, postoperatively reversal of low SMI is not an indicator of better prognosis, contrasting previous studies reported that increased SMI could reduce recurrence and death risk [14, 36]. Our observations revealed that the reversal of low SMI was associated with a higher risk of poor RFS at 9 months postoperatively but not at 3, 6 or 12 months postoperatively. This relationship may be due to the sample size and patient characteristics, as those with low preoperative and high postoperative SMI at 9 months were older, had a lower BMI and had more advanced (T4) tumours (all *p* < 0.05). Future prospective studies with larger sample sizes are needed to explore whether the postoperative reversal of low SMI could improve CRC prognosis.

Our study narrowed this window to 1 year after surgery because it was a critical period characterized by dynamic physiological changes, the stabilization of body weight and the most notable loss in muscle mass, which significantly impact patient recovery and outcomes [13, 18]. Monitoring these changes is crucial for understanding the effects of surgical intervention on overall health and recovery. Furthermore, current guidelines recommend that patients with stage II and III CRC undergo chest and abdominal CT imaging every 6–12 months [37]. This aligns with our study period, ensuring that our research is consistent with established medical practices. By focusing on this timeframe, our findings have the potential to be integrated into routine clinical practice, allowing for the quantification of body composition from regularly acquired CT images [38]. This could significantly enhance patient care by providing healthcare professionals with additional tools to monitor and manage patient recovery and health post‐surgery. Additionally, this time frame is also consistent with the study periods in the existing literature [36], which facilitates comparative analysis and enhances the robustness of our findings. By using a similar timeframe, our study can directly compare its results with those of previous research, providing a more comprehensive understanding of SMI changes and their impact on patient prognosis.

Our study's findings have significant clinical implications for the perioperative management of CRC patients. The identification of distinct SMI change patterns provides a framework for tailoring interventions based on individual patient needs. Postoperative SMI can be easily measured by routine follow‐up CT scans without increased dose of radiation nor economic burden. Hence, routine monitoring of SMI status should be performed to guide patient care. Future prospective studies should aim to validate these findings and investigate targeted interventions for each pattern to improve long‐term outcomes.

Research indicates that skeletal muscle in perioperative period can be modified by interventions such as physical exercise, nutritional supplementation, hormone replacement and therapeutic agents [39]. [S1–S4] (Data S1), which may improve the prognosis. Based on our study observations, we recommend initiating interventions aimed at mitigating muscle mass loss within the first 3 months postoperatively. Although patients are recovering from a significant procedure like a laparotomy, this period also presents a unique window for intervention. Studies indicate that structured physical activity, especially resistance training and aerobic exercises, can significantly help in preserving lean mass post‐surgery. For example, rehabilitation programmes involving progressive strength training, aerobic training and balance exercises have shown improvements in muscle strength and physical function in patients recovering from surgeries such as hip fractures [S5]. Additionally, addressing inflammation and malnutrition immediately post‐surgery can pre‐emptively mitigate the immune and metabolic dysfunction that leads to muscle wasting in cancer patients [28]. Enhanced Recovery After Surgery (ERAS) protocols also recommend measures such as early oral feeding and early mobilization to reduce physiological stress and psychological pressure on surgical patients, thereby accelerating their postoperative recovery [S6]. However, the ongoing recovery process must be carefully managed to balance the benefits of these interventions with the patient's capacity to tolerate them. Future trials are also needed to evaluate the feasibility and effectiveness of these interventions in preventing or reversing within postoperative 1‐year low SMI and improving survival of CRC patients.

It should be noted that the cut‐off values of low SMI in this study are lower than those of previous studies (38.5 cm^2^/m^2^ in women and 52.4 cm^2^/m^2^ in men) [5, 15] in that the population of our study have a lower BMI than that in other countries [S7–S9]. However, the cut‐off value showed similar trends as those of other studies [4, 5, 14, 15], including male cut‐off value is higher than that of females and Caucasian cut‐off value is higher than that of Asians [16] [S10]. In this context, establishing our own low SMI cut‐off value plays a crucial role in intervening skeletal muscle status and may provide references for similar clinical practice in other regions. Future studies are needed to validate these cut‐off values and explore their implications in different populations.

It is worth noting that our study has several limitations. Firstly, it is a retrospective study, which may introduce bias and limit causal inferences. Secondly, the study was conducted at a single centre, which may limit the generalizability of the findings. Thirdly, we were unable to account for other clinically relevant potential covariates, including neoadjuvant radiation, steroid exposure, concomitant medications, caregiver and/or marital status, education level and clinical laboratory values related to inflammation. Unfortunately, functional assessments such as muscle strength were not available from the medical records, which represents a limitation of our study. Nevertheless, recent definitions of sarcopenia recognize low muscle mass as a primary criterion for probable sarcopenia. Our study's findings have significant clinical implications for the perioperative management of CRC patients, underscoring the clinical importance of managing muscle mass in this patient population.

In conclusion, our study highlights the importance of assessing skeletal muscle status in CRC patients, as perioperative SMI change is an independent prognostic factor for RFS and OS. Patients experiencing postoperative low SMI present the poorest prognosis, but the reversal of low SMI not a risk factor of recurrence and death. Patients with persistently low SMI are also significantly associated with poor OS. Monitoring SMI changes during the perioperative period may serve as a useful tool for risk stratification and treatment decision‐making in CRC patients. Further prospective studies with larger sample sizes are warranted to validate these findings and explore potential interventions to prevent or reverse low SMI in CRC patients.

## Conflict of Interest

None reported.

## Supporting information


**Figure S1** Representative axial CT images show the four change patterns in skeletal muscle index in patients. Quantitative CT of skeletal muscle (red) at baseline and at 3‐month, 6‐month, 9‐month, and 12‐month postoperative. (a) Male, 75 years, high_pre_‐high_post_, BMI = 25.46; (b) Male, 67 years, high_pre_‐low_post_, BMI = 19.83; (c) Male, 64 years, low_pre_‐high_post_, BMI = 21.30; (d) Male,71 years, low_pre_‐low_post_, BMI = 20.80.

**Figure S2** Kaplan–Meier curves for recurrence‐free survival (RFS) of low and high skeletal muscle index (SMI) groups. Statistical significance was calculated by the log‐rank test: (a) at postoperative 3 months, (b) at postoperative 6 months, (c) at postoperative 9 months, (d) at postoperative 12 months

**Figure S3** Kaplan–Meier curves for overall survival (OS) of low and high skeletal muscle index (SMI) groups. Statistical significance was calculated by the log‐rank test: (a) at postoperative 3 months, (b) at postoperative 6 months, (c) at postoperative 9 months, (d) at postoperative 12 months.

**Figure S4** Hazard ratio trend by univariate analysis with postoperative follow‐up time. Reference group: high_pre_‐high_post_; Orange: high_pre_‐low_post_; blue: low_pre_‐high_post_; red: low_pre_‐low_post_. (a) RFS, recurrence‐free survival; (b) OS, overall survival.

**Data S1** Supporting Information

**Table S1.** The CT image acquisition parameters.
**Table S2.** Comparison of demographic characteristics of Included and excluded populations.
**Table S3.** Demographic and clinicopathological baseline characteristics of the population with and without postoperative CT scan data.
**Table S4.** Demographic and clinicopathological Characteristics Categorized by Skeletal Muscle Changes at postoperative 3 months.
**Table S5.** Demographic and clinicopathological Characteristics Categorized by Skeletal Muscle Changes at postoperative 6 months.
**Table S6.** Demographic and clinicopathological Characteristics Categorized by Skeletal Muscle Changes at postoperative 9 months.
**Table S7.** Demographic and clinicopathological Characteristics Categorized by Skeletal Muscle Changes at postoperative 12 months.
**Table S8.** Univariate and multivariate analysis of skeletal muscle index and recurrence free survival at preoperative baseline, postoperative 3, 6, 9, and 12 months.
**Table S9.** Univariate and multivariate analysis of skeletal muscle index and overall survival at preoperative baseline, postoperative 3, 6, 9, and 12 months.
