# Supplementary figures and images for: Association of Perioperative Skeletal Muscle Index Change With Outcome in Colorectal Cancer Patients
Source: J Cachexia Sarcopenia Muscle. 2024 Oct 3;15(6):2519–35. doi: 10.1002/jcsm.13594 (PMC11634468; doi:10.1002/jcsm.13594)

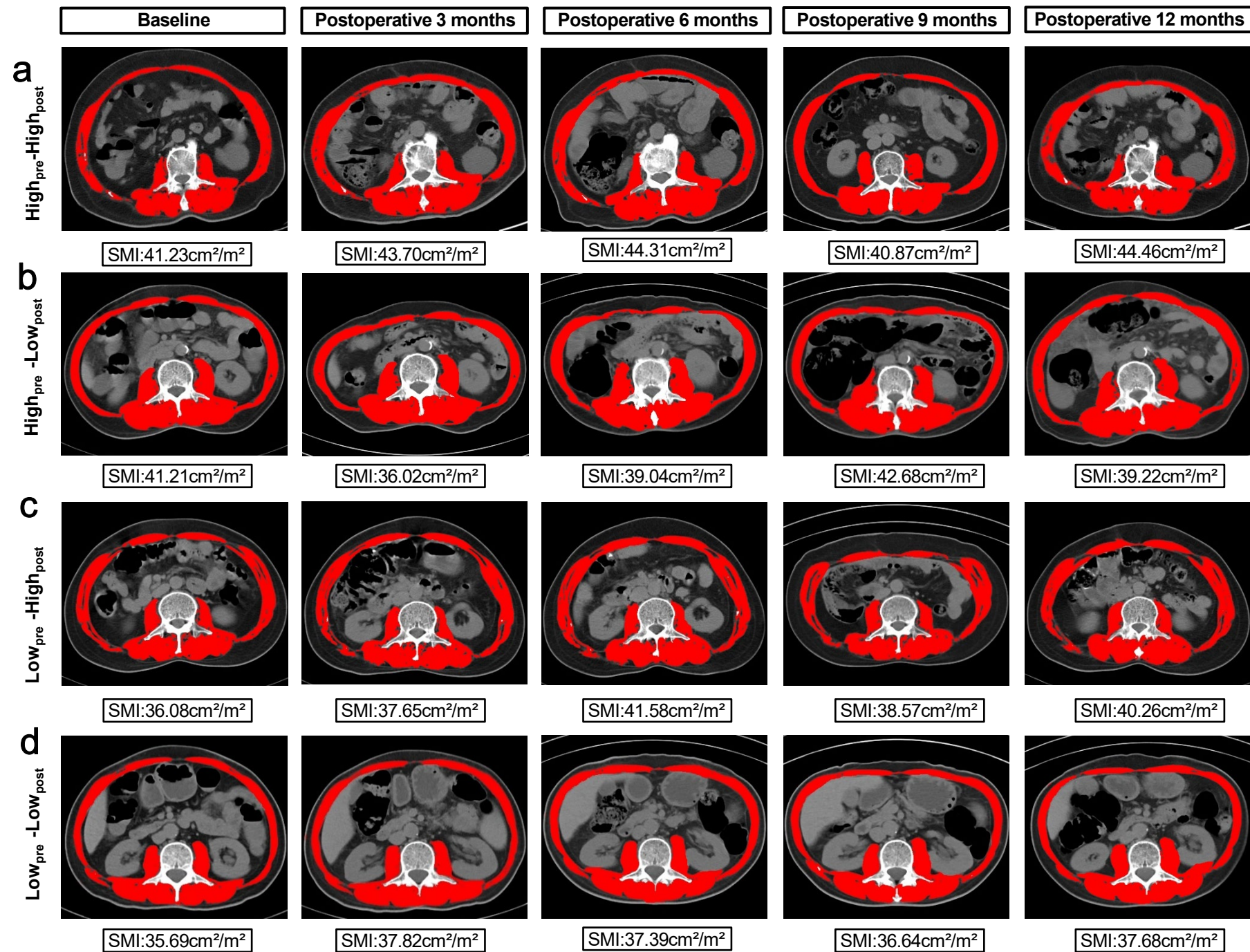

Supplement: Supplementary file 1 — Figure S1 Representative axial CT images show the four change patterns in skeletal muscle index in patients. Quantitative CT of skeletal muscle (red) at baseline and at 3‐month, 6‐month, 9‐month, and 12‐month postoperative. (a) Male, 75 years, highpre‐highpost, BMI = 25.46; (b) Male, 67 years, highpre‐lowpost, BMI = 19.83; (c) Male, 64 years, lowpre‐highpost, BMI = 21.30; (d) Male,71 years, lowpre‐lowpost, BMI = 20.80. [file JCSM-15-2519-s002.pdf]

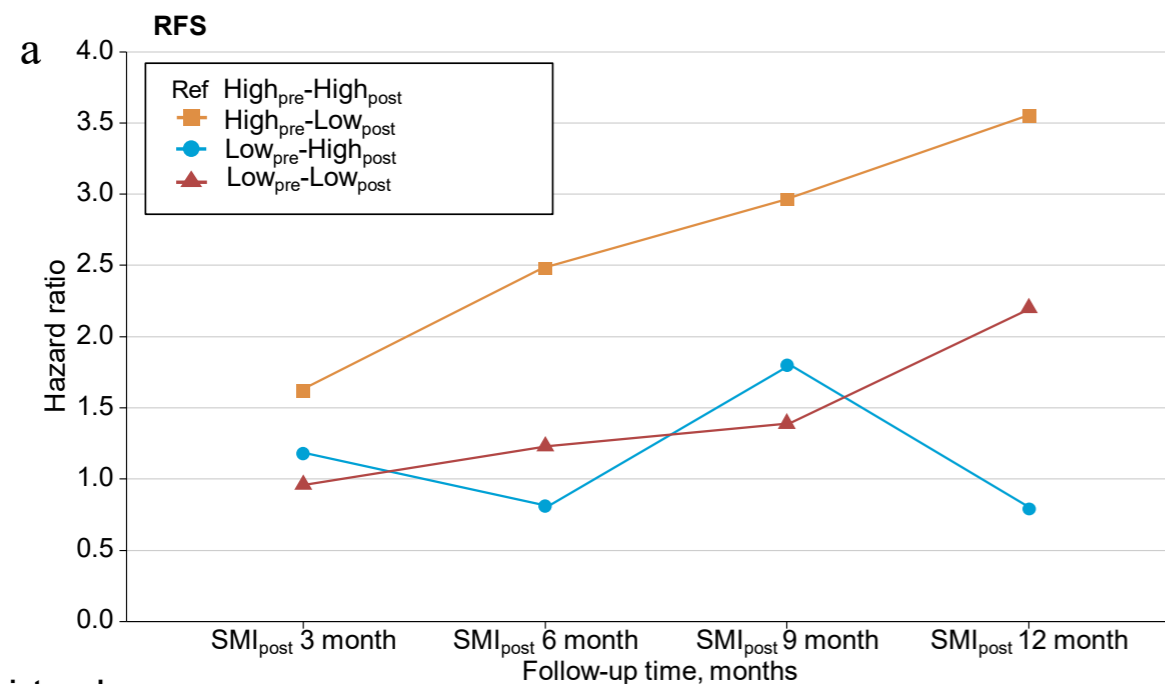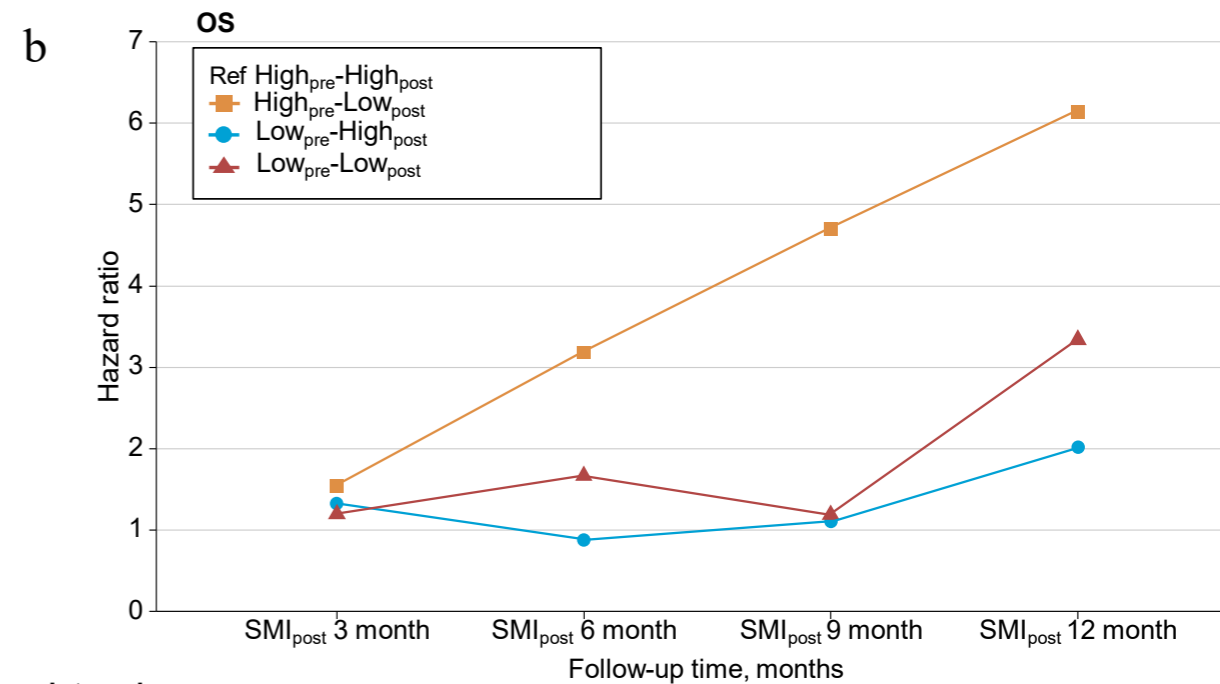

Supplement: Supplementary file 4 — Figure S4 Hazard ratio trend by univariate analysis with postoperative follow‐up time. Reference group: highpre‐highpost; Orange: highpre‐lowpost; blue: lowpre‐highpost; red: lowpre‐lowpost. (a) RFS, recurrence‐free survival; (b) OS, overall survival. [file JCSM-15-2519-s001.pdf]
