# Supplementary material for: Association of Perioperative Skeletal Muscle Index Change With Outcome in Colorectal Cancer Patients
Source: J Cachexia Sarcopenia Muscle. 2024 Oct 3;15(6):2519–35. doi: 10.1002/jcsm.13594 (PMC11634468; doi:10.1002/jcsm.13594)
Supplement: Supplementary file 2 — Figure S2 Kaplan–Meier curves for recurrence‐free survival (RFS) of low and high skeletal muscle index (SMI) groups. Statistical significance was calculated by the log‐rank test: (a) at postoperative 3 months, (b) at postoperative 6 months, (c) at postoperative 9 months, (d) at postoperative 12 months [file JCSM-15-2519-s003.pdf]

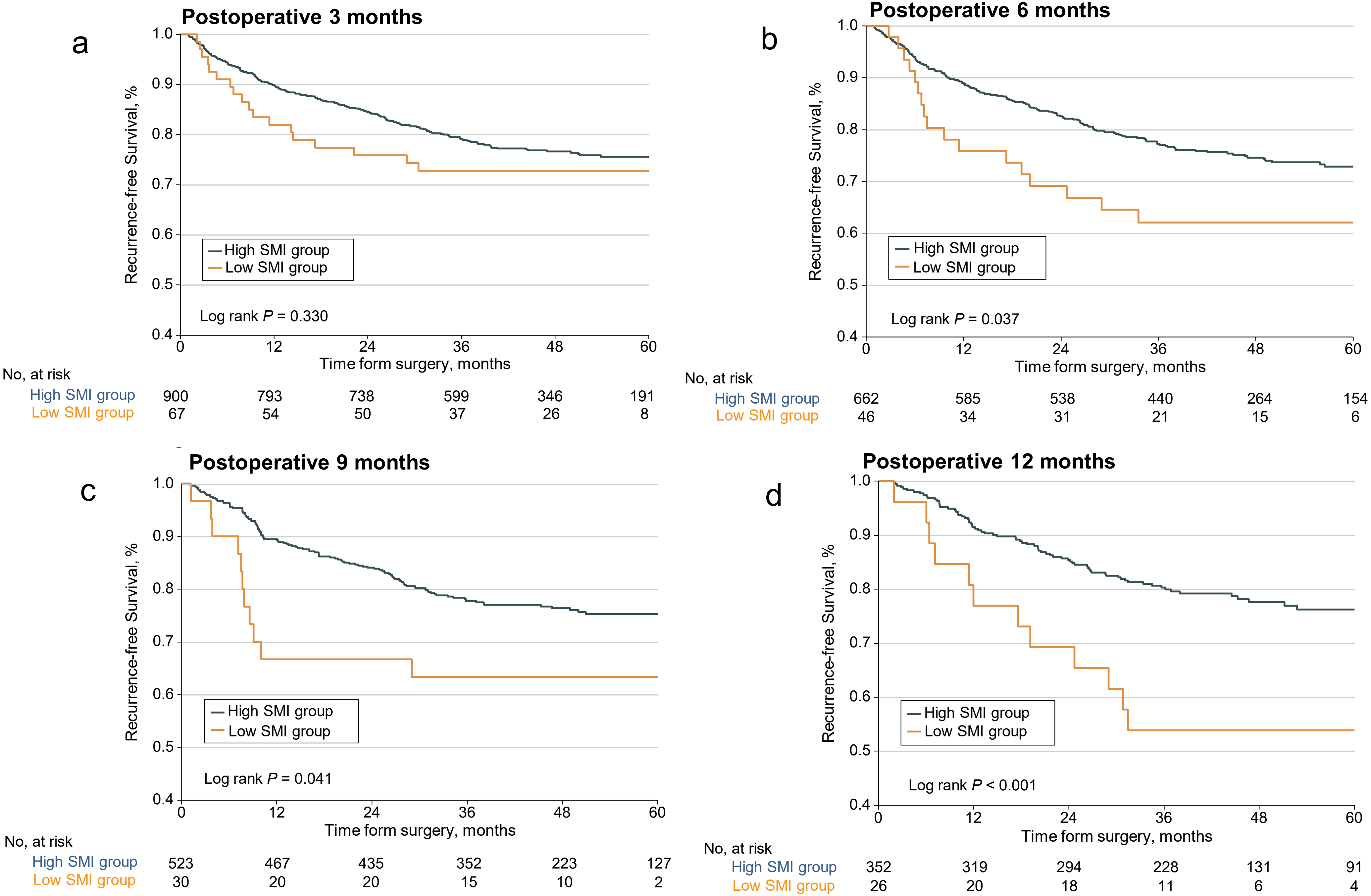
a
Postoperative 3 months
Recurrence-free Survival, %
1.0
0.9
0.8
0.7
0.6
0.5
0.4
High SMI group
Low SMI group
Log rank P = 0.330
0
12
24
36
48
60
Time form surgery, months
No, at risk
High SMI group 900 793 738 599 346 191
Low SMI group 67 54 50 37 26 8
b
Postoperative 6 months
Recurrence-free Survival, %
High SMI group
Low SMI group
Log rank P = 0.037
Time form surgery, months
No, at risk
High SMI group 662 585 538 440 264 154
Low SMI group 46 34 31 21 15 6
c
Postoperative 9 months
Recurrence-free Survival, %
High SMI group
Low SMI group
Log rank P = 0.041
Time form surgery, months
No, at risk
High SMI group 523 467 435 352 223 127
Low SMI group 30 20 20 15 10 2
d
Postoperative 12 months
Recurrence-free Survival, %
High SMI group
Low SMI group
Log rank P < 0.001
Time form surgery, months
No, at risk
High SMI group 352 319 294 228 131 91
Low SMI group 26 20 18 11 6 4
