# Supplementary material for: Association of Perioperative Skeletal Muscle Index Change With Outcome in Colorectal Cancer Patients
Source: J Cachexia Sarcopenia Muscle. 2024 Oct 3;15(6):2519–35. doi: 10.1002/jcsm.13594 (PMC11634468; doi:10.1002/jcsm.13594)
Supplement: Supplementary file 3 — Figure S3 Kaplan–Meier curves for overall survival (OS) of low and high skeletal muscle index (SMI) groups. Statistical significance was calculated by the log‐rank test: (a) at postoperative 3 months, (b) at postoperative 6 months, (c) at postoperative 9 months, (d) at postoperative 12 months. [file JCSM-15-2519-s006.pdf]

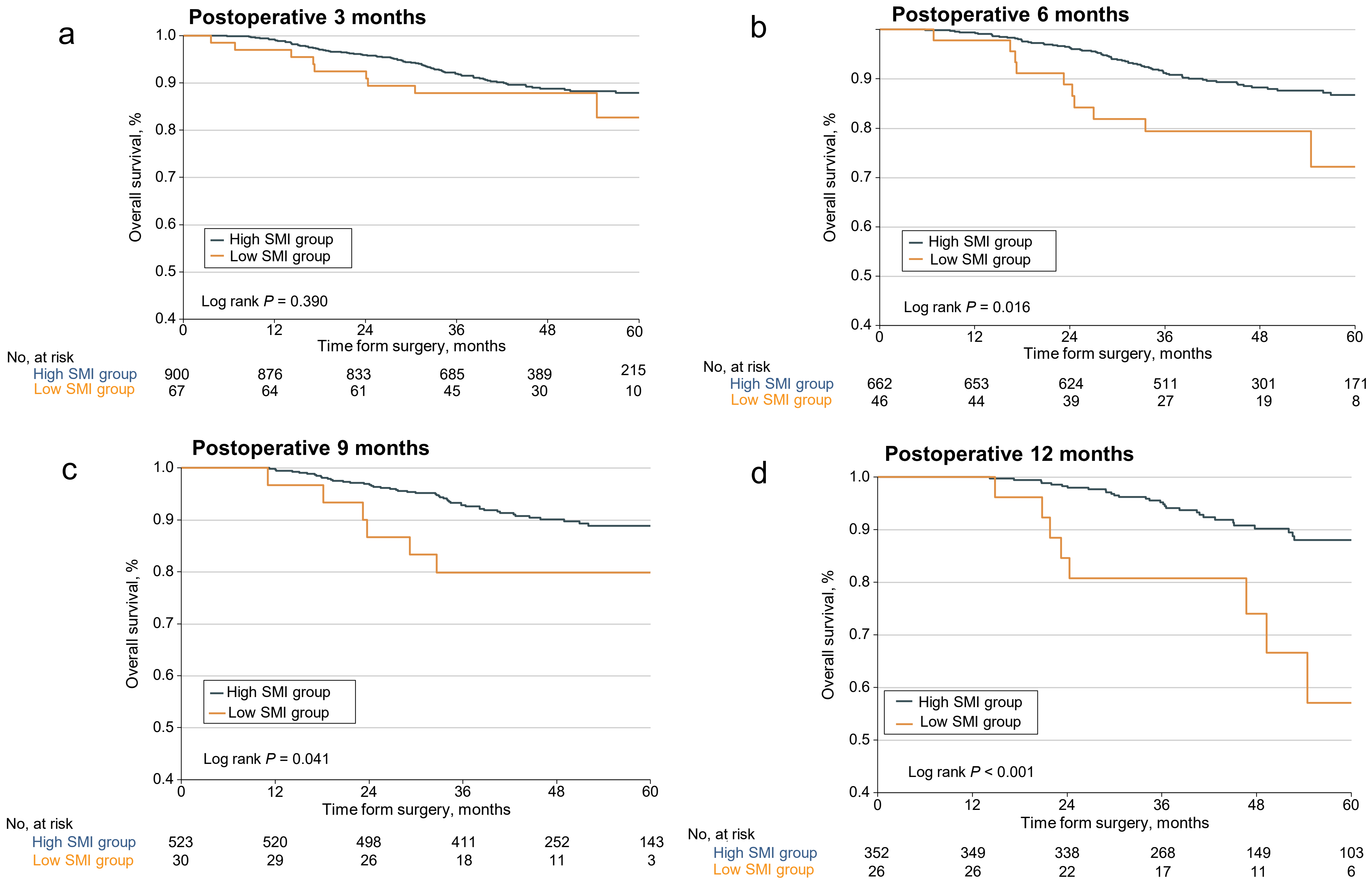
a
Postoperative 3 months
Overall survival, %
1.0
0.9
0.8
0.7
0.6
0.5
0.4
High SMI group
Low SMI group
Log rank P = 0.390
0
12
24
36
48
60
Time form surgery, months
No, at risk
High SMI group 900 876 833 685 389 215
Low SMI group 67 64 61 45 30 10
b
Postoperative 6 months
Overall survival, %
0.9
0.8
0.7
0.6
0.5
0.4
High SMI group
Low SMI group
Log rank P = 0.016
0
12
24
36
48
60
Time form surgery, months
No, at risk
High SMI group 662 653 624 511 301 171
Low SMI group 46 44 39 27 19 8
c
Postoperative 9 months
Overall survival, %
1.0
0.9
0.8
0.7
0.6
0.5
0.4
High SMI group
Low SMI group
Log rank P = 0.041
0
12
24
36
48
60
Time form surgery, months
No, at risk
High SMI group 523 520 498 411 252 143
Low SMI group 30 29 26 18 11 3
d
Postoperative 12 months
Overall survival, %
1.0
0.9
0.8
0.7
0.6
0.5
0.4
High SMI group
Low SMI group
Log rank P < 0.001
0
12
24
36
48
60
Time form surgery, months
No, at risk
High SMI group 352 349 338 268 149 103
Low SMI group 26 26 22 17 11 6
