# Supplementary material for: Association of Perioperative Skeletal Muscle Index Change With Outcome in Colorectal Cancer Patients
Source: J Cachexia Sarcopenia Muscle. 2024 Oct 3;15(6):2519–35. doi: 10.1002/jcsm.13594 (PMC11634468; doi:10.1002/jcsm.13594)
Supplement: Supplementary file 5 — Data S1 Supporting Information [file JCSM-15-2519-s005.docx]

**Supplementary references**

S1. Javed AA, Mayhew AJ, Shea AK, Raina P. Association Between Hormone Therapy and Muscle Mass in Postmenopausal Women: A Systematic Review and Meta-analysis. *JAMA Netw Open* 2019;2:e1910154.

S2. Dutt V, Gupta S, Dabur R, Injeti E, Mittal A. Skeletal muscle atrophy: Potential therapeutic agents and their mechanisms of action. *Pharmacol Res* 2015;99:86–100.

S3. Stene GB, Helbostad JL, Balstad TR, Riphagen II, Kaasa S, Oldervoll LM. Effect of physical exercise on muscle mass and strength in cancer patients during treatment—A systematic review. *Critical Reviews in Oncology/Hematology* 2013;88:573–593.

S4. Martin-Cantero A, Reijnierse EM, Gill BMT, Maier AB. Factors influencing the efficacy of nutritional interventions on muscle mass in older adults: a systematic review and meta-analysis. *Nutr Rev* 2021;79:315–330.

S5. Phang JK, Lim ZY, Yee WQ, Tan CYF, Kwan YH, Low LL. Post-surgery interventions for hip fracture: a systematic review of randomized controlled trials. BMC Musculoskelet Disord 2023;24.

S6. Gustafsson UO, Scott MJ, Hubner M, Nygren J, Demartines N, Francis N *et al.* Guidelines for Perioperative Care in Elective Colorectal Surgery: Enhanced Recovery After Surgery (ERAS^®^) Society Recommendations: 2018. *World j surg* 2018;43:659–695.

S7. Deurenberg P, Deurenberg-Yap M, Guricci S. Asians are different from Caucasians and from each other in their body mass index/body fat per cent relationship. Obes Rev 2002;3:141–6.

S8. Wang J, Thornton JC, Russell M, Burastero S, Heymsfield S, Pierson RN Jr. Asians have lower body mass index (BMI) but higher percent body fat than do whites: comparisons of anthropometric measurements. Am J Clin Nutr 1994;60:23–8.

S9. WHO Expert Consultation. Appropriate body-mass index for Asian populations and its implications for policy and intervention strategies. Lancet 2004;363:157–63.

S10. Lau EMC, Lynn HSH, Woo JW, Kwok TCY, Melton LJ 3rd. Prevalence of and risk factors for low SMI in elderly Chinese men and women. J Gerontol A Biol Sci Med Sci 2005;60:213–6.
