# Supplementary material for: Association of Perioperative Skeletal Muscle Index Change With Outcome in Colorectal Cancer Patients
Source: J Cachexia Sarcopenia Muscle. 2024 Oct 3;15(6):2519–35. doi: 10.1002/jcsm.13594 (PMC11634468; doi:10.1002/jcsm.13594)
Supplement: Supplementary file 6 — Table S1. The CT image acquisition parameters. Table S2. Comparison of demographic characteristics of Included and excluded populations. Table S3. Demographic and clinicopathological baseline characteristics of the population with and without postoperative CT scan data. Table S4. Demographic and clinicopathological Characteristics Categorized by Skeletal Muscle Changes at postoperative 3 months. Table S5. Demographic and clinicopathological Characteristics Categorized by Skeletal Muscle Changes at postoperative 6 months. Table S6. Demographic and clinicopathological Characteristics Categorized by Skeletal Muscle Changes at postoperative 9 months. Table S7. Demographic and clinicopathological Characteristics Categorized by Skeletal Muscle Changes at postoperative 12 months. Table S8. Univariate and multivariate analysis of skeletal muscle index and recurrence free survival at preoperative baseline, postoperative 3, 6, 9, and 12 months. Table S9. Univariate and multivariate analysis of skeletal muscle index and overall survival at preoperative baseline, postoperative 3, 6, 9, and 12 months. [file JCSM-15-2519-s004.docx]

**Supplementary Table**

Table S1. The CT image acquisition parameters

Table S2. Comparison of demographic characteristics of Included and excluded populations

Table S3. Demographic and clinicopathological baseline characteristics of the population with and without postoperative CT scan data

Table S4. Demographic and clinicopathological Characteristics Categorized by Skeletal Muscle Changes at postoperative 3 months

Table S5. Demographic and clinicopathological Characteristics Categorized by Skeletal Muscle Changes at postoperative 6 months

Table S6. Demographic and clinicopathological Characteristics Categorized by Skeletal Muscle Changes at postoperative 9 months

Table S7. Demographic and clinicopathological Characteristics Categorized by Skeletal Muscle Changes at postoperative 12 months

Table S8. Univariate and multivariate analysis of skeletal muscle index and recurrence free survival at preoperative baseline, postoperative 3, 6, 9, and 12 months.

Table S9. Univariate and multivariate analysis of skeletal muscle index and overall survival at preoperative baseline, postoperative 3, 6, 9, and 12 months.

# **Table S1. The CT image acquisition parameters**

| **Parameters** | **128-slice spiral CT 1** | **128-slice spiral CT 2** |
| --- | --- | --- |
| CT system | SOMATOM Deﬁnition AS+, Siemens Healthineers | PHILIPS Brilliance  128-slice CT scanner |
| Contrast | infused 1.5 ml/kg of the non-ionic contrast material (Iohexol, 300 mgI/ml Omnipaque; GE Healthcare) at a rate of 3.5 ml/s | infused 1.5 ml/kg of the non-ionic contrast material (Iohexol, 300 mgI/ml Omnipaque; GE Healthcare) at a rate of 3.5 ml/s |
| portal venous phase  Time | 65-75 s after injection | 65-75 s after injection |
| Tube voltage | 120 kVp | 120 kVp |
| Tube current | 290-330 mA | 290-330 mA |
| Rotation time | 0.50 s | 0.50 s |
| Detector collimation | 128×0.6 mm | 128×0.6 mm |
|  |  |  |
| Image matrix | 512×512 | 512×512 |
| Field of view | 500×500 mm | 500×500 mm |
| Reconstruction section thickness | 1mm-2 mm | 1mm-2 mm |

# **Table S2. Comparison of demographic characteristics of Included and excluded populations**

| **Parameter** | | **All Patients^1^**  **(n = 2580)** | **Excluded^1^**  **(n = 358)** | **Included^1^**  **(n = 2222)** | ***P* value^2^** |
| --- | --- | --- | --- | --- | --- |
| Age, year | |  |  |  |  |
| Median (IQR) | | 60.00 (51.00, 67.25) | 59.00 (51.00, 66.75) | 60.00 (51.00, 68.00) | 0.741 |
| Sex, n (%) | |  |  |  | 0.071 |
| Female | | 1087 (42.13) | 167 (46.65) | 920 (41.40) |  |
| Male | | 1493 (57.87) | 191 (53.35) | 1302 (58.60) |  |
| BMI, (kg/m2) | |  |  |  |  |
| Median (IQR) | | 22.49 (20.76, 24.89) | 22.12 (20.54, 24.60) | 22.59 (20.76, 24.92) | 0.050 |
| Smoking history, n (%) | |  |  |  | 0.604 |
| Yes | | 641 (24.84) | 85 (23.74) | 556 (25.02) |  |
| No | | 1912 (74.11) | 268 (74.86) | 1644 (73.99) |  |
| Unknow | | 27 (1.05) | 5 (1.40) | 22 (0.99) |  |
| Drinking history, n (%) | |  |  |  | 0.340 |
| Yes | | 477 (18.49) | 58 (16.20) | 419 (18.86) |  |
| No | | 2014 (78.06) | 290 (81.01) | 1724 (77.59) |  |
| Unknow | | 89 (3.45) | 10 (2.79) | 79 (3.56) |  |
| Hypertension, n (%) | |  |  |  | 0.290 |
| Yes | | 622 (24.11) | 87 (24.30) | 535 (24.08) |  |
| No | | 1948 (75.50) | 268 (74.86) | 1680 (75.61) |  |
| Unknow | | 10 (0.39) | 3 (0.84) | 7 (0.32) |  |
| Diabetes, n (%) | |  |  |  | 0.427 |
| Yes | | 211 (8.18) | 23 (6.42) | 188 (8.46) |  |
| No | | 2355 (91.28) | 333 (93.02) | 2022 (91.00) |  |
| Unknow | | 14 (0.54) | 2 (0.56) | 12 (0.54) |  |
| Coronary heart disease, n (%) | |  |  |  | 0.599 |
| Yes | | 72 (2.79) | 7 (1.96) | 65 (2.93) |  |
| No | | 2496 (96.74) | 349 (97.49) | 2147 (96.62) |  |
| Unknow | | 12 (0.47) | 2 (0.56) | 10 (0.45) |  |
| COPD, n (%) | |  |  |  | 1.000 |
| Yes | | 24 (0.93) | 3 (0.84) | 21 (0.95) |  |
| No | | 2534 (98.22) | 352 (98.32) | 2182 (98.20) |  |
| Unknow | | 22 (0.85) | 3 (0.84) | 19 (0.86) |  |
| ECOG grade, n (%) | |  |  |  | 0.706 |
| 0 | | 1338 (51.86) | 178 (49.72) | 1160 (52.21) |  |
| 1 | | 1121 (43.45) | 160 (44.69) | 961 (43.25) |  |
| 2 | | 74 (2.87) | 13 (3.63) | 61 (2.75) |  |
| ≥3 | | 47 (1.82) | 7 (1.96) | 40 (1.80) |  |
| Weight change at postoperative 3 months, (kg) | | |  |  |  |
| Median (IQR) | | -3.00 (-5.00, -1.00) | -3.00 (-5.00, 0.00) | -3.00 (-5.00, -1.00) | 0.018 |
| Weight change at postoperative 3 months, n (%) | | |  |  | 0.116 |
| Decline | | 1419 (55.00) | 192 (53.63) | 1227 (55.22) |  |
| Increase | | 458 (17.75) | 77 (21.51) | 381 (17.15) |  |
| Unknown | | 703 (27.25) | 89 (24.86) | 614 (27.63) |  |
| Preoperative serum albumin, (g/L) | |  |  |  |  |
| Median (IQR) | | 45.00 (41.90, 47.00) | 43.75 (41.00, 46.45) | 45.00 (42.00, 47.00) | <0.001 |
| Preoperative serum albumin group, n (%) | |  |  |  | 0.034 |
| <35 | | 91 (3.53) | 5 (1.40) | 86 (3.87) |  |
| ≥35 | | 2485 (96.32) | 353 (98.60) | 2132 (95.95) |  |
| Unknown | | 4 (0.16) | 0 (0.00) | 4 (0.18) |  |
| Primary site, n (%) | |  |  |  | 0.853 |
| Colon | | 1226 (47.52) | 168 (46.93) | 1058 (47.61) |  |
| Rectum | | 1354 (52.48) | 190 (53.07) | 1164 (52.39) |  |
| Pathological stage, n (%) | |  |  |  | <0.001 |
| I | | 602 (23.33) | 74 (20.67) | 528 (23.76) |  |
| II | | 1026 (39.77) | 179 (50.00) | 847 (38.12) |  |
| III | | 952 (36.90) | 105 (29.33) | 847 (38.12) |  |
| Tumor differentiation, n (%) |  | |  |  | 0.216 |
| Well+ Moderate | | 716 (27.75) | 113 (31.56) | 603 (27.14) |  |
| Poor | | 1644 (63.72) | 215 (60.06) | 1429 (64.31) |  |
| Unknown | | 220 (8.53) | 30 (8.38) | 190 (8.55) |  |
| Histologic type, n (%) | |  |  |  | 0.725 |
| Mucinous type | | 2429 (94.15) | 339 (94.69) | 2090 (94.06) |  |
| Non–Mucinous type | | 151 (5.85) | 19 (5.31) | 132 (5.94) |  |
| T stage, n (%) | |  |  |  | 0.028 |
| T1 | | 217 (8.41) | 31 (8.66) | 186 (8.37) |  |
| T2 | | 477 (18.49) | 53 (14.80) | 424 (19.08) |  |
| T3 | | 1760 (68.22) | 247 (68.99) | 1513 (68.09) |  |
| T4 | | 126 (4.88) | 27 (7.54) | 99 (4.46) |  |
| N stage, n (%) | |  |  |  | 0.018 |
| N0 | | 1604 (62.17) | 246 (68.72) | 1358 (61.12) |  |
| N1 | | 700 (27.13) | 77 (21.51) | 623 (28.04) |  |
| N2 | | 276 (10.70) | 35 (9.78) | 241 (10.85) |  |
| Lymph node yield, n (%) | |  |  |  | 0.201 |
| <12 | | 543 (21.05) | 85 (23.74) | 458 (20.61) |  |
| ≥12 | | 2037 (78.95) | 273 (76.26) | 1764 (79.39) |  |
| Lymph vascular invasion, n (%) | |  |  |  | 0.176 |
| Yes | | 202 (7.83) | 28 (7.82) | 174 (7.83) |  |
| No | | 394 (15.27) | 43 (12.01) | 351 (15.80) |  |
| Unknown | | 1984 (76.90) | 287 (80.17) | 1697 (76.37) |  |
| Perineural invasion, n (%) | |  |  |  | 0.055 |
| Yes | | 59 (2.29) | 5 (1.40) | 54 (2.43) |  |
| No | | 503 (19.50) | 56 (15.64) | 447 (20.12) |  |
| Unknown | | 2018 (78.22) | 297 (82.96) | 1721 (77.45) |  |
| Tumor deposit, n (%) | |  |  |  | 0.020 |
| Yes | | 223 (8.64) | 19 (5.31) | 204 (9.18) |  |
| No | | 2357 (91.36) | 339 (94.69) | 2018 (90.82) |  |
| Adjuvant chemotherapy, n (%) | |  |  |  | 0.876 |
| Yes | | 1464 (56.74) | 205 (57.26) | 1259 (56.66) |  |
| No | | 1116 (43.26) | 153 (42.74) | 963 (43.34) |  |

Note:

^1^n (%); Median (IQR)

^2^Pearson's Chi-squared test; Wilcoxon rank sum test; Fisher's exact test

Abbreviations: IQR, interquartile range; SD, standard deviation

# **Table S3.** **Demographic and clinicopathological baseline characteristics of the population with and without postoperative CT scan data**

| **Parameter** | | **With postoperative data^1^**  **(n = 1237)** | **Without postoperative data^1^**  **(n = 985)** | ***P*-Value^2^** |
| --- | --- | --- | --- | --- |
| Age, year | |  |  |  |
| Median (IQR) | | 59.00 (51.00, 66.00) | 62.00 (52.00, 69.00) | <0.001 |
| Sex, n (%) | |  |  | 0.452 |
| Female | | 503 (40.66) | 417 (42.34) |  |
| Male | | 734 (59.34) | 568 (57.66) |  |
| BMI, (kg/m^2^) | |  |  |  |
| Median (IQR) | | 22.72 (20.76, 25.11) | 22.33 (20.57, 24.61) | 0.001 |
| Smoking history, n (%) | |  |  | 0.469 |
| Yes | | 322 (26.03) | 234 (23.76) |  |
| No | | 903 (73.00) | 741 (75.23) |  |
| Unknow | | 12 (0.97) | 10 (1.02) |  |
| Drinking history, n (%) | |  |  | 0.211 |
| Yes | | 249 (20.13) | 170 (17.26) |  |
| No | | 943 (76.23) | 781 (79.29) |  |
| Unknow | | 45 (3.64) | 34 (3.45) |  |
| Hypertension, n (%) | |  |  | 0.617 |
| Yes | | 307 (24.82) | 228 (23.15) |  |
| No | | 926 (74.86) | 754 (76.55) |  |
| Unknow | | 4 (0.32) | 3 (0.30) |  |
| Diabetes, n (%) | |  |  | 0.981 |
| Yes | | 105 (8.49) | 83 (8.43) |  |
| No | | 1125 (90.95) | 897 (91.07) |  |
| Unknow | | 7 (0.57) | 5 (0.51) |  |
| Coronary heart disease, n (%) | |  |  | 0.336 |
| Yes | | 42 (3.40) | 23 (2.34) |  |
| No | | 1190 (96.20) | 957 (97.16) |  |
| Unknow | | 5 (0.40) | 5 (0.51) |  |
| COPD, n (%) | |  |  | 0.128 |
| Yes | | 8 (0.65) | 13 (1.32) |  |
| No | | 1221 (98.71) | 961 (97.56) |  |
| Unknow | | 8 (0.65) | 11 (1.12) |  |
| ECOG grade, n (%) | |  |  | <0.001 |
| 0 | | 702 (56.75) | 458 (46.50) |  |
| 1 | | 509 (41.15) | 452 (45.89) |  |
| 2 | | 16 (1.29) | 45 (4.57) |  |
| ≥3 | | 10 (0.81) | 30 (3.05) |  |
| Weight change at postoperative 3 months, (kg) | | |  |  |
| Median (IQR) | | -3.00 (-5.00, -1.00) | -3.00 (-5.00, 0.00) | 0.051 |
| Weight change at postoperative 3 months, n (%) | | |  | <0.001 |
| Decline | | 787 (63.62) | 440 (44.67) |  |
| Increase | | 216 (17.46) | 165 (16.75) |  |
| Unknown | | 234 (18.92) | 380 (38.58) |  |
| Preoperative serum albumin, (g/L) | |  |  |  |
| Median (IQR) | | 46.00 (43.00, 48.00) | 44.00 (40.55, 46.00) | <0.001 |
| Preoperative serum albumin group, n (%) | |  |  | 0.031 |
| <35 | | 37 (2.99) | 49 (4.97) |  |
| ≥35 | | 1197 (96.77) | 935 (94.92) |  |
| Unknown | | 3 (0.24) | 1 (0.10) |  |
| Primary site, n (%) | |  |  | 0.578 |
| Colon | | 596 (48.18) | 462 (46.90) |  |
| Rectum | | 641 (51.82) | 523 (53.10) |  |
| Pathological stage, n (%) | |  |  | 0.875 |
| I | | 299 (24.17) | 229 (23.25) |  |
| II | | 468 (37.83) | 379 (38.48) |  |
| III | | 470 (38.00) | 377 (38.27) |  |
| Tumor differentiation, n (%) |  | |  | 0.003 |
| Well+ Moderate | | 310 (25.06) | 293 (29.75) |  |
| Poor | | 833 (67.34) | 596 (60.51) |  |
| Unknown | | 94 (7.60) | 96 (9.75) |  |
| Histologic type, n (%) | |  |  | 0.001 |
| Mucinous type | | 1144 (92.48) | 946 (96.04) |  |
| Non–Mucinous type | | 93 (7.52) | 39 (3.96) |  |
| T stage, n (%) | |  |  | 0.037 |
| T1 | | 103 (8.33) | 83 (8.43) |  |
| T2 | | 244 (19.73) | 180 (18.27) |  |
| T3 | | 822 (66.45) | 691 (70.15) |  |
| T4 | | 68 (5.50) | 31 (3.15) |  |
| N stage, n (%) | |  |  | 0.298 |
| N0 | | 761 (61.52) | 597 (60.61) |  |
| N1 | | 353 (28.54) | 270 (27.41) |  |
| N2 | | 123 (9.94) | 118 (11.98) |  |
| Lymph node yield, n (%) | |  |  | <0.001 |
| <12 | | 199 (16.09) | 259 (26.29) |  |
| ≥12 | | 1038 (83.91) | 726 (73.71) |  |
| Lymph vascular invasion, n (%) | |  |  | 0.017 |
| Yes | | 100 (8.08) | 74 (7.51) |  |
| No | | 171 (13.82) | 180 (18.27) |  |
| Unknown | | 966 (78.09) | 731 (74.21) |  |
| Perineural invasion, n (%) | |  |  | 0.014 |
| Yes | | 23 (1.86) | 31 (3.15) |  |
| No | | 230 (18.59) | 217 (22.03) |  |
| Unknown | | 984 (79.55) | 737 (74.82) |  |
| Tumor deposit, n (%) | |  |  | <0.001 |
| Yes | | 146 (11.80) | 58 (5.89) |  |
| No | | 1091 (88.20) | 927 (94.11) |  |
| Adjuvant chemotherapy, n (%) | |  |  | <0.001 |
| Yes | | 803 (64.92) | 456 (46.29) |  |
| No | | 434 (35.08) | 529 (53.71) |  |

Note:

^1^n (%); Median (IQR)

^2^Pearson's Chi-squared test; Wilcoxon rank sum test; Fisher's exact test

Abbreviations: IQR, interquartile range; SD, standard deviation.

# **Table S4. Demographic and Clinicopathological Characteristics Categorized by Skeletal Muscle Changes at postoperative 3 months**

| **Parameter** | **All Patients^1^**  **(n = 967)** | **Skeletal muscle changes at postoperative 3 months** | | | | ***P*-Value^2^** |
| --- | --- | --- | --- | --- | --- | --- |
|  |  | **High_pre_-High_post_^1^**  **(n = 865)** | **High_pre_-Low_post_^1^**  **(n = 34)** | **Low_pre_-High_post_^1^**  **(n = 35)** | **Low_pre_-Low_post_^1^**  **(n = 33)** |  |
| Age, year |  |  |  |  |  |  |
| Median (IQR) | 59.00 (51.00, 66.00) | 58.00 (50.00, 65.00) | 63.50 (55.25, 66.75) | 63.00 (54.50, 71.50) | 65.00 (61.00, 71.00) | <0.001 |
| Sex,n (%) |  |  |  |  |  | 0.049 |
| Female | 394 (40.74) | 365 (42.20) | 11 (32.35) | 8 (22.86) | 10 (30.30) |  |
| Male | 573 (59.26) | 500 (57.80) | 23 (67.65) | 27 (77.14) | 23 (69.70) |  |
| BMI, (kg/m2) |  |  |  |  |  |  |
| Median (IQR) | 22.76 (20.76, 25.21) | 23.23 (21.11, 25.39) | 21.84 (20.76, 24.49) | 20.76 (19.17, 21.48) | 20.76 (18.42, 20.94) | <0.001 |
| Smoking history, n (%) |  |  |  |  |  | 0.021 |
| Yes | 261 (26.99) | 226 (26.13) | 14 (41.18) | 10 (28.57) | 11 (33.33) |  |
| No | 698 (72.18) | 634 (73.29) | 19 (55.88) | 23 (65.71) | 22 (66.67) |  |
| Unknow | 8 (0.83) | 5 (0.58) | 1 (2.94) | 2 (5.71) | 0 (0.00) |  |
| Drinking history, n (%) |  |  |  |  |  | 0.201 |
| Yes | 199 (20.58) | 173 (20.00) | 9 (26.47) | 9 (25.71) | 8 (24.24) |  |
| No | 728 (75.28) | 659 (76.18) | 21 (61.76) | 24 (68.57) | 24 (72.73) |  |
| Unknow | 40 (4.14) | 33 (3.82) | 4 (11.76) | 2 (5.71) | 1 (3.03) |  |
| Hypertension, n (%) |  |  |  |  |  | 0.281 |
| Yes | 243 (25.13) | 215 (24.86) | 10 (29.41) | 11 (31.43) | 7 (21.21) |  |
| No | 721 (74.56) | 648 (74.91) | 24 (70.59) | 23 (65.71) | 26 (78.79) |  |
| Unknow | 3 (0.31) | 2 (0.23) | 0 (0.00) | 1 (2.86) | 0 (0.00) |  |
| Diabetes, n (%) |  |  |  |  |  | 0.132 |
| Yes | 79 (8.17) | 69 (7.98) | 5 (14.71) | 3 (8.57) | 2 (6.06) |  |
| No | 882 (91.21) | 792 (91.56) | 28 (82.35) | 31 (88.57) | 31 (93.94) |  |
| Unknow | 6 (0.62) | 4 (0.46) | 1 (2.94) | 1 (2.86) | 0 (0.00) |  |
| Coronary heart disease, n (%) |  |  |  |  |  | 0.280 |
| Yes | 30 (3.10) | 27 (3.12) | 2 (5.88) | 1 (2.86) | 0 (0.00) |  |
| No | 933 (96.48) | 835 (96.53) | 32 (94.12) | 33 (94.29) | 33 (100.00) |  |
| Unknow | 4 (0.41) | 3 (0.35) | 0 (0.00) | 1 (2.86) | 0 (0.00) |  |
| COPD, n (%) |  |  |  |  |  | 0.245 |
| Yes | 5 (0.52) | 4 (0.46) | 0 (0.00) | 0 (0.00) | 1 (3.03) |  |
| No | 957 (98.97) | 857 (99.08) | 34 (100.00) | 34 (97.14) | 32 (96.97) |  |
| Unknow | 5 (0.52) | 4 (0.46) | 0 (0.00) | 1 (2.86) | 0 (0.00) |  |
| ECOG grade, n (%) |  |  |  |  |  | 0.914 |
| 0 | 552 (57.08) | 495 (57.23) | 19 (55.88) | 20 (57.14) | 18 (54.55) |  |
| 1 | 397 (41.05) | 354 (40.92) | 15 (44.12) | 14 (40.00) | 14 (42.42) |  |
| 2 | 10 (1.03) | 8 (0.92) | 0 (0.00) | 1 (2.86) | 1 (3.03) |  |
| ≥3 | 8 (0.83) | 8 (0.92) | 0 (0.00) | 0 (0.00) | 0 (0.00) |  |
| Weight change at postoperative 3 months, (kg) | |  |  |  |  |  |
| Median (IQR) | -3.00 (-5.00, -1.00) | -3.00 (-5.00, -1.00) | -6.00 (-10.00, -2.00) | -1.20 (-4.00, 1.00) | -2.00 (-3.50, -0.50) | <0.001 |
| Weight change at postoperative 3 months, n (%) | |  |  |  |  | 0.002 |
| Decline | 621 (64.22) | 551 (63.70) | 31 (91.18) | 16 (45.71) | 23 (69.70) |  |
| Increase | 169 (17.48) | 148 (17.11) | 2 (5.88) | 11 (31.43) | 8 (24.24) |  |
| Unknown | 177 (18.30) | 166 (19.19) | 1 (2.94) | 8 (22.86) | 2 (6.06) |  |
| Preoperative serum albumin, (g/L) |  |  |  |  |  |  |
| Median (IQR) | 46.00 (43.00, 48.00) | 46.00 (43.00, 48.00) | 46.00 (44.25, 48.00) | 45.00 (40.50, 47.50) | 44.00 (41.00, 48.00) | 0.158 |
| Preoperative serum albumin group, n (%) | |  |  |  |  | 0.410 |
| <35 | 31 (3.21) | 26 (3.01) | 1 (2.94) | 3 (8.57) | 1 (3.03) |  |
| ≥35 | 934 (96.59) | 837 (96.76) | 33 (97.06) | 32 (91.43) | 32 (96.97) |  |
| Unknown | 2 (0.21) | 2 (0.23) | 0 (0.00) | 0 (0.00) | 0 (0.00) |  |
| Primary site, No. (%) |  |  |  |  |  | 0.381 |
| Colon | 469 (48.50) | 420 (48.55) | 14 (41.18) | 21 (60.00) | 14 (42.42) |  |
| Rectum | 498 (51.50) | 445 (51.45) | 20 (58.82) | 14 (40.00) | 19 (57.58) |  |
| Pathological stage, No. (%) |  |  |  |  |  | 0.766 |
| I | 227 (23.47) | 202 (23.35) | 7 (20.59) | 7 (20.00) | 11 (33.33) |  |
| II | 375 (38.78) | 332 (38.38) | 14 (41.18) | 16 (45.71) | 13 (39.39) |  |
| III | 365 (37.75) | 331 (38.27) | 13 (38.24) | 12 (34.29) | 9 (27.27) |  |
| Tumor differentiation, No. (%) |  |  |  |  |  | 0.329 |
| Well+ Moderate | 235 (24.30) | 213 (24.62) | 5 (14.71) | 8 (22.86) | 9 (27.27) |  |
| Poor | 665 (68.77) | 590 (68.21) | 29 (85.29) | 23 (65.71) | 23 (69.70) |  |
| Unknown | 67 (6.93) | 62 (7.17) | 0 (0.00) | 4 (11.43) | 1 (3.03) |  |
| Histologic type, No. (%) |  |  |  |  |  | 0.613 |
| Mucinous type | 886 (91.62) | 792 (91.56) | 33 (97.06) | 31 (88.57) | 30 (90.91) |  |
| Non–Mucinous type | 81 (8.38) | 73 (8.44) | 1 (2.94) | 4 (11.43) | 3 (9.09) |  |
| T stage, No. (%) |  |  |  |  |  | 0.023 |
| T1 | 71 (7.34) | 68 (7.86) | 0 (0.00) | 2 (5.71) | 1 (3.03) |  |
| T2 | 192 (19.86) | 169 (19.54) | 7 (20.59) | 5 (14.29) | 11 (33.33) |  |
| T3 | 647 (66.91) | 580 (67.05) | 27 (79.41) | 22 (62.86) | 18 (54.55) |  |
| T4 | 57 (5.89) | 48 (5.55) | 0 (0.00) | 6 (17.14) | 3 (9.09) |  |
| N stage, No. (%) |  |  |  |  |  | 0.507 |
| N0 | 597 (61.74) | 529 (61.16) | 21 (61.76) | 23 (65.71) | 24 (72.73) |  |
| N1 | 274 (28.34) | 247 (28.55) | 8 (23.53) | 11 (31.43) | 8 (24.24) |  |
| N2 | 96 (9.93) | 89 (10.29) | 5 (14.71) | 1 (2.86) | 1 (3.03) |  |
| Lymph node yield, No. (%) |  |  |  |  |  | 0.305 |
| <12 | 157 (16.24) | 139 (16.07) | 5 (14.71) | 4 (11.43) | 9 (27.27) |  |
| ≥12 | 810 (83.76) | 726 (83.93) | 29 (85.29) | 31 (88.57) | 24 (72.73) |  |
| Lymph vascular invasion, No. (%) |  |  |  |  |  | 0.595 |
| Yes | 78 (8.07) | 74 (8.55) | 1 (2.94) | 1 (2.86) | 2 (6.06) |  |
| No | 141 (14.58) | 129 (14.91) | 2 (5.88) | 5 (14.29) | 5 (15.15) |  |
| Unknown | 748 (77.35) | 662 (76.53) | 31 (91.18) | 29 (82.86) | 26 (78.79) |  |
| Perineural invasion, No. (%) |  |  |  |  |  | 0.756 |
| Yes | 20 (2.07) | 20 (2.31) | 0 (0.00) | 0 (0.00) | 0 (0.00) |  |
| No | 183 (18.92) | 168 (19.42) | 3 (8.82) | 6 (17.14) | 6 (18.18) |  |
| Unknown | 764 (79.01) | 677 (78.27) | 31 (91.18) | 29 (82.86) | 27 (81.82) |  |
| Tumor deposit, No. (%) |  |  |  |  |  | 0.402 |
| Yes | 122 (12.62) | 108 (12.49) | 6 (17.65) | 6 (17.14) | 2 (6.06) |  |
| No | 845 (87.38) | 757 (87.51) | 28 (82.35) | 29 (82.86) | 31 (93.94) |  |
| Adjuvant chemotherapy, No. (%) |  |  |  |  |  | 0.688 |
| Yes | 646 (66.80) | 577 (66.71) | 24 (70.59) | 21 (60.00) | 24 (72.73) |  |
| No | 321 (33.20) | 288 (33.29) | 10 (29.41) | 14 (40.00) | 9 (27.27) |  |

Note：

^1^n (%); Median (IQR)

^2^Pearson's Chi-squared test; Wilcoxon rank sum test; Fisher's exact test

Abbreviations: IQR, interquartile range; SD, standard deviation; COPD, Chronic Obstructive Pulmonary Disease; ECOG, Eastern Cooperative Oncology Group

# **Table S5. Demographic and Clinicopathological Characteristics Categorized by Skeletal Muscle Changes at postoperative 6 months**

| **Parameter** | **All Patients^1^**  **(n = 708)** | **Skeletal muscle changes at postoperative 6 months** | | | | ***P*-Value^2^** |
| --- | --- | --- | --- | --- | --- | --- |
|  |  | **High_pre_-High_post_^1^**  **(n = 635)** | **High_pre_-Low_post_^1^**  **(n = 18)** | **Low_pre_-High_post_^1^**  **(n = 27)** | **Low_pre_-Low_post_^1^**  **(n = 28)** |  |
| Age, year |  |  |  |  |  |  |
| Median (IQR) | 58.00 (50.00, 65.00) | 57.00 (49.00, 64.00) | 65.00 (56.50, 69.75) | 63.00 (57.00, 71.50) | 66.50 (61.75, 71.75) | <0.001 |
| Sex, No. (%) |  |  |  |  |  | 0.610 |
| Female | 263 (37.15) | 241 (37.95) | 6 (33.33) | 8 (29.63) | 8 (28.57) |  |
| Male | 445 (62.85) | 394 (62.05) | 12 (66.67) | 19 (70.37) | 20 (71.43) |  |
| BMI, (kg/m^2^) |  |  |  |  |  |  |
| Median (IQR) | 22.83 (20.76, 25.22) | 23.24 (21.10, 25.39) | 21.26 (20.76, 23.77) | 20.76 (20.51, 22.20) | 19.95 (18.40, 20.87) | <0.001 |
| Smoking history, n (%) |  |  |  |  |  | 0.463 |
| Yes | 197 (27.82) | 173 (27.24) | 6 (33.33) | 9 (33.33) | 9 (32.14) |  |
| No | 504 (71.19) | 456 (71.81) | 11 (61.11) | 18 (66.67) | 19 (67.86) |  |
| Unknow | 7 (0.99) | 6 (0.94) | 1 (5.56) | 0 (0.00) | 0 (0.00) |  |
| Drinking history, n (%) |  |  |  |  |  | 0.936 |
| Yes | 153 (21.61) | 136 (21.42) | 4 (22.22) | 6 (22.22) | 7 (25.00) |  |
| No | 530 (74.86) | 476 (74.96) | 13 (72.22) | 20 (74.07) | 21 (75.00) |  |
| Unknow | 25 (3.53) | 23 (3.62) | 1 (5.56) | 1 (3.70) | 0 (0.00) |  |
| Hypertension, n (%) |  |  |  |  |  | 0.915 |
| Yes | 172 (24.29) | 154 (24.25) | 4 (22.22) | 8 (29.63) | 6 (21.43) |  |
| No | 534 (75.42) | 479 (75.43) | 14 (77.78) | 19 (70.37) | 22 (78.57) |  |
| Unknow | 2 (0.28) | 2 (0.31) | 0 (0.00) | 0 (0.00) | 0 (0.00) |  |
| Diabetes, n (%) |  |  |  |  |  | 0.300 |
| Yes | 66 (9.32) | 57 (8.98) | 4 (22.22) | 4 (14.81) | 1 (3.57) |  |
| No | 639 (90.25) | 575 (90.55) | 14 (77.78) | 23 (85.19) | 27 (96.43) |  |
| Unknow | 3 (0.42) | 3 (0.47) | 0 (0.00) | 0 (0.00) | 0 (0.00) |  |
| Coronary heart disease, n (%) |  |  |  |  |  | 1.000 |
| Yes | 22 (3.11) | 21 (3.31) | 0 (0.00) | 0 (0.00) | 1 (3.57) |  |
| No | 685 (96.75) | 613 (96.54) | 18 (100.00) | 27 (100.00) | 27 (96.43) |  |
| Unknow | 1 (0.14) | 1 (0.16) | 0 (0.00) | 0 (0.00) | 0 (0.00) |  |
| COPD, n (%) |  |  |  |  |  | 0.583 |
| Yes | 5 (0.71) | 4 (0.63) | 0 (0.00) | 0 (0.00) | 1 (3.57) |  |
| No | 700 (98.87) | 628 (98.90) | 18 (100.00) | 27 (100.00) | 27 (96.43) |  |
| Unknow | 3 (0.42) | 3 (0.47) | 0 (0.00) | 0 (0.00) | 0 (0.00) |  |
| ECOG grade, n (%) |  |  |  |  |  | 0.709 |
| 0 | 387 (54.66) | 347 (54.65) | 9 (50.00) | 19 (70.37) | 12 (42.86) |  |
| 1 | 305 (43.08) | 273 (42.99) | 9 (50.00) | 8 (29.63) | 15 (53.57) |  |
| 2 | 10 (1.41) | 9 (1.42) | 0 (0.00) | 0 (0.00) | 1 (3.57) |  |
| ≥3 | 6 (0.85) | 6 (0.94) | 0 (0.00) | 0 (0.00) | 0 (0.00) |  |
| Weight change at postoperative 3 months, (kg) | |  |  |  |  |  |
| Median (IQR) | -3.00 (-5.00, -1.00) | -3.00 (-5.00, -1.00) | -6.00 (-8.00, -2.00) | -2.00 (-3.25, 0.00) | -2.00 (-4.00, -1.00) | 0.004 |
| Weight change at postoperative 3 months, n (%) | |  |  |  |  | 0.419 |
| Decline | 486 (68.64) | 436 (68.66) | 16 (88.89) | 16 (59.26) | 18 (64.29) |  |
| Increase | 127 (17.94) | 113 (17.80) | 1 (5.56) | 8 (29.63) | 5 (17.86) |  |
| Unknown | 95 (13.42) | 86 (13.54) | 1 (5.56) | 3 (11.11) | 5 (17.86) |  |
| Preoperative serum albumin, (g/L) |  |  |  |  |  |  |
| Median (IQR) | 46.00 (43.00, 48.00) | 46.00 (43.00, 48.00) | 46.50 (44.25, 49.00) | 45.00 (43.00, 48.00) | 45.50 (43.00, 47.25) | 0.738 |
| Preoperative serum albumin group, n (%) | |  |  |  |  | 0.420 |
| <35 | 23 (3.25) | 19 (2.99) | 1 (5.56) | 1 (3.70) | 2 (7.14) |  |
| ≥35 | 683 (96.47) | 614 (96.69) | 17 (94.44) | 26 (96.30) | 26 (92.86) |  |
| Unknown | 2 (0.28) | 2 (0.31) | 0 (0.00) | 0 (0.00) | 0 (0.00) |  |
| Primary site, n (%) |  |  |  |  |  | 0.418 |
| Colon | 362 (51.13) | 330 (51.97) | 7 (38.89) | 14 (51.85) | 11 (39.29) |  |
| Rectum | 346 (48.87) | 305 (48.03) | 11 (61.11) | 13 (48.15) | 17 (60.71) |  |
| Pathological stage, n (%) |  |  |  |  |  | 0.409 |
| I | 123 (17.37) | 105 (16.54) | 3 (16.67) | 6 (22.22) | 9 (32.14) |  |
| II | 289 (40.82) | 259 (40.79) | 9 (50.00) | 10 (37.04) | 11 (39.29) |  |
| III | 296 (41.81) | 271 (42.68) | 6 (33.33) | 11 (40.74) | 8 (28.57) |  |
| Tumor differentiation, n (%) |  |  |  |  |  | 0.778 |
| Well+ Moderate | 182 (25.71) | 167 (26.30) | 3 (16.67) | 4 (14.81) | 8 (28.57) |  |
| Poor | 476 (67.23) | 422 (66.46) | 14 (77.78) | 22 (81.48) | 18 (64.29) |  |
| Unknown | 50 (7.06) | 46 (7.24) | 1 (5.56) | 1 (3.70) | 2 (7.14) |  |
| Histologic type, n (%) |  |  |  |  |  | 0.615 |
| Mucinous type | 649 (91.67) | 579 (91.18) | 18 (100.00) | 25 (92.59) | 27 (96.43) |  |
| Non–Mucinous type | 59 (8.33) | 56 (8.82) | 0 (0.00) | 2 (7.41) | 1 (3.57) |  |
| T stage, n (%) |  |  |  |  |  | 0.876 |
| T1 | 41 (5.79) | 36 (5.67) | 1 (5.56) | 1 (3.70) | 3 (10.71) |  |
| T2 | 108 (15.25) | 95 (14.96) | 2 (11.11) | 5 (18.52) | 6 (21.43) |  |
| T3 | 521 (73.59) | 469 (73.86) | 15 (83.33) | 19 (70.37) | 18 (64.29) |  |
| T4 | 38 (5.37) | 35 (5.51) | 0 (0.00) | 2 (7.41) | 1 (3.57) |  |
| N stage, n (%) |  |  |  |  |  | 0.108 |
| N0 | 408 (57.63) | 361 (56.85) | 12 (66.67) | 16 (59.26) | 19 (67.86) |  |
| N1 | 227 (32.06) | 201 (31.65) | 6 (33.33) | 11 (40.74) | 9 (32.14) |  |
| N2 | 73 (10.31) | 73 (11.50) | 0 (0.00) | 0 (0.00) | 0 (0.00) |  |
| Lymph node yield, n (%) |  |  |  |  |  | 0.258 |
| <12 | 113 (15.96) | 96 (15.12) | 4 (22.22) | 7 (25.93) | 6 (21.43) |  |
| ≥12 | 595 (84.04) | 539 (84.88) | 14 (77.78) | 20 (74.07) | 22 (78.57) |  |
| Lymph vascular invasion, n (%) |  |  |  |  |  | 0.500 |
| Yes | 69 (9.75) | 66 (10.39) | 0 (0.00) | 1 (3.70) | 2 (7.14) |  |
| No | 85 (12.01) | 77 (12.13) | 3 (16.67) | 4 (14.81) | 1 (3.57) |  |
| Unknown | 554 (78.25) | 492 (77.48) | 15 (83.33) | 22 (81.48) | 25 (89.29) |  |
| Perineural invasion, n (%) |  |  |  |  |  | 0.961 |
| Yes | 14 (1.98) | 14 (2.20) | 0 (0.00) | 0 (0.00) | 0 (0.00) |  |
| No | 126 (17.80) | 115 (18.11) | 3 (16.67) | 5 (18.52) | 3 (10.71) |  |
| Unknown | 568 (80.23) | 506 (79.69) | 15 (83.33) | 22 (81.48) | 25 (89.29) |  |
| Tumor deposit, n (%) |  |  |  |  |  | 0.419 |
| Yes | 95 (13.42) | 87 (13.70) | 3 (16.67) | 4 (14.81) | 1 (3.57) |  |
| No | 613 (86.58) | 548 (86.30) | 15 (83.33) | 23 (85.19) | 27 (96.43) |  |
| Adjuvant chemotherapy, n (%) |  |  |  |  |  | 0.414 |
| Yes | 531 (75.00) | 480 (75.59) | 12 (66.67) | 21 (77.78) | 18 (64.29) |  |
| No | 177 (25.00) | 155 (24.41) | 6 (33.33) | 6 (22.22) | 10 (35.71) |  |

Note：

^1^n (%); Median (IQR)

^2^Pearson's Chi-squared test; Wilcoxon rank sum test; Fisher's exact test

Abbreviations: IQR, interquartile range; SD, standard deviation; COPD, Chronic Obstructive Pulmonary Disease; ECOG, Eastern Cooperative Oncology Group

# **Table S6. Demographic and Clinicopathological Characteristics Categorized by Skeletal Muscle Changes at postoperative 9 months**

| **Parameter** | **All Patients^1^**  **(n = 553)** | **Skeletal muscle changes at postoperative 9 months** | | | | ***P*-Value^2^** |
| --- | --- | --- | --- | --- | --- | --- |
|  |  | **High_pre_-High_post_^1^**  **(n = 498)** | **High_pre_-Low_post_^1^**  **(n = 12)** | **Low_pre_-High_post_^1^**  **(n = 25)** | **Low_pre_-Low_post_^1^**  **(n = 18)** |  |
| Age, year |  |  |  |  |  |  |
| Median (IQR) | 58.00 (50.00, 64.00) | 57.00 (50.00, 63.75) | 61.50 (54.00, 70.25) | 65.00 (51.00, 71.00) | 66.00 (59.50, 71.00) | 0.003 |
| Sex, n (%) |  |  |  |  |  | 0.048 |
| Female | 230 (41.59) | 212 (42.57) | 6 (50.00) | 4 (16.00) | 8 (44.44) |  |
| Male | 323 (58.41) | 286 (57.43) | 6 (50.00) | 21 (84.00) | 10 (55.56) |  |
| BMI, (kg/m^2^) |  |  |  |  |  |  |
| Median (IQR) | 22.83 (20.80, 25.34) | 23.23 (21.10, 25.59) | 23.05 (21.81, 25.61) | 20.28 (18.07, 21.72) | 20.76 (17.92, 21.03) | <0.001 |
| Smoking history, n (%) |  |  |  |  |  | 0.068 |
| Yes | 144 (26.04) | 126 (25.30) | 3 (25.00) | 11 (44.00) | 4 (22.22) |  |
| No | 402 (72.69) | 367 (73.69) | 8 (66.67) | 13 (52.00) | 14 (77.78) |  |
| Unknow | 7 (1.27) | 5 (1.00) | 1 (8.33) | 1 (4.00) | 0 (0.00) |  |
| Drinking history, n (%) |  |  |  |  |  | 0.402 |
| Yes | 115 (20.80) | 101 (20.28) | 2 (16.67) | 8 (32.00) | 4 (22.22) |  |
| No | 416 (75.23) | 378 (75.90) | 9 (75.00) | 15 (60.00) | 14 (77.78) |  |
| Unknow | 22 (3.98) | 19 (3.82) | 1 (8.33) | 2 (8.00) | 0 (0.00) |  |
| Hypertension, n (%) |  |  |  |  |  | 0.098 |
| Yes | 143 (25.86) | 126 (25.30) | 6 (50.00) | 7 (28.00) | 4 (22.22) |  |
| No | 408 (73.78) | 371 (74.50) | 6 (50.00) | 17 (68.00) | 14 (77.78) |  |
| Unknow | 2 (0.36) | 1 (0.20) | 0 (0.00) | 1 (4.00) | 0 (0.00) |  |
| Diabetes, n (%) |  |  |  |  |  | 0.068 |
| Yes | 46 (8.32) | 38 (7.63) | 3 (25.00) | 4 (16.00) | 1 (5.56) |  |
| No | 503 (90.96) | 457 (91.77) | 9 (75.00) | 20 (80.00) | 17 (94.44) |  |
| Unknow | 4 (0.72) | 3 (0.60) | 0 (0.00) | 1 (4.00) | 0 (0.00) |  |
| Coronary heart disease, n (%) |  |  |  |  |  | 0.229 |
| Yes | 20 (3.62) | 17 (3.41) | 1 (8.33) | 1 (4.00) | 1 (5.56) |  |
| No | 529 (95.66) | 478 (95.98) | 11 (91.67) | 23 (92.00) | 17 (94.44) |  |
| Unknow | 4 (0.72) | 3 (0.60) | 0 (0.00) | 1 (4.00) | 0 (0.00) |  |
| COPD, n (%) |  |  |  |  |  | 0.409 |
| Yes | 2 (0.36) | 2 (0.40) | 0 (0.00) | 0 (0.00) | 0 (0.00) |  |
| No | 548 (99.10) | 494 (99.20) | 12 (100.00) | 24 (96.00) | 18 (100.00) |  |
| Unknow | 3 (0.54) | 2 (0.40) | 0 (0.00) | 1 (4.00) | 0 (0.00) |  |
| ECOG grade, n (%) |  |  |  |  |  | 0.121 |
| 0 | 305 (55.15) | 275 (55.22) | 7 (58.33) | 17 (68.00) | 6 (33.33) |  |
| 1 | 236 (42.68) | 213 (42.77) | 4 (33.33) | 8 (32.00) | 11 (61.11) |  |
| 2 | 8 (1.45) | 7 (1.41) | 0 (0.00) | 0 (0.00) | 1 (5.56) |  |
| ≥3 | 4 (0.72) | 3 (0.60) | 1 (8.33) | 0 (0.00) | 0 (0.00) |  |
| Weight change at postoperative 3 months, (kg) | |  |  |  |  |  |
| Median (IQR) | -3.00 (-5.00, -1.00) | -3.00 (-5.00, -1.00) | -6.00 (-10.00, -1.25) | -1.00 (-4.00, 1.00) | -1.50 (-3.00, -1.00) | 0.019 |
| Weight change at postoperative 3 months, n (%) | |  |  |  |  | 0.086 |
| Decline | 371 (67.09) | 336 (67.47) | 9 (75.00) | 12 (48.00) | 14 (77.78) |  |
| Increase | 96 (17.36) | 81 (16.27) | 2 (16.67) | 9 (36.00) | 4 (22.22) |  |
| Unknown | 86 (15.55) | 81 (16.27) | 1 (8.33) | 4 (16.00) | 0 (0.00) |  |
| Preoperative serum albumin, (g/L) |  |  |  |  |  |  |
| Median (IQR) | 46.00 (43.00, 48.25) | 46.00 (43.00, 49.00) | 46.50 (44.50, 48.25) | 45.00 (42.00, 47.00) | 46.50 (44.00, 48.00) | 0.348 |
| Preoperative serum albumin group, n (%) | |  |  |  |  | 0.273 |
| <35 | 15 (2.71) | 12 (2.41) | 0 (0.00) | 2 (8.00) | 1 (5.56) |  |
| ≥35 | 537 (97.11) | 485 (97.39) | 12 (100.00) | 23 (92.00) | 17 (94.44) |  |
| Unknown | 1 (0.18) | 1 (0.20) | 0 (0.00) | 0 (0.00) | 0 (0.00) |  |
| Primary site, n (%) |  |  |  |  |  | 0.714 |
| Colon | 272 (49.19) | 243 (48.80) | 6 (50.00) | 15 (60.00) | 8 (44.44) |  |
| Rectum | 281 (50.81) | 255 (51.20) | 6 (50.00) | 10 (40.00) | 10 (55.56) |  |
| Pathological stage, n (%) |  |  |  |  |  | 0.727 |
| I | 105 (18.99) | 96 (19.28) | 1 (8.33) | 5 (20.00) | 3 (16.67) |  |
| II | 210 (37.97) | 187 (37.55) | 4 (33.33) | 9 (36.00) | 10 (55.56) |  |
| III | 238 (43.04) | 215 (43.17) | 7 (58.33) | 11 (44.00) | 5 (27.78) |  |
| Tumor differentiation, n (%) |  |  |  |  |  | 0.955 |
| Well+ Moderate | 141 (25.50) | 127 (25.50) | 4 (33.33) | 5 (20.00) | 5 (27.78) |  |
| Poor | 373 (67.45) | 335 (67.27) | 7 (58.33) | 19 (76.00) | 12 (66.67) |  |
| Unknown | 39 (7.05) | 36 (7.23) | 1 (8.33) | 1 (4.00) | 1 (5.56) |  |
| Histologic type, n (%) |  |  |  |  |  | 0.403 |
| Mucinous type | 504 (91.14) | 453 (90.96) | 12 (100.00) | 24 (96.00) | 15 (83.33) |  |
| Non–Mucinous type | 49 (8.86) | 45 (9.04) | 0 (0.00) | 1 (4.00) | 3 (16.67) |  |
| T stage, n (%) |  |  |  |  |  | 0.015 |
| T1 | 26 (4.70) | 25 (5.02) | 0 (0.00) | 1 (4.00) | 0 (0.00) |  |
| T2 | 108 (19.53) | 100 (20.08) | 1 (8.33) | 4 (16.00) | 3 (16.67) |  |
| T3 | 388 (70.16) | 349 (70.08) | 11 (91.67) | 14 (56.00) | 14 (77.78) |  |
| T4 | 31 (5.61) | 24 (4.82) | 0 (0.00) | 6 (24.00) | 1 (5.56) |  |
| N stage, n (%) |  |  |  |  |  | 0.720 |
| N0 | 314 (56.78) | 282 (56.63) | 5 (41.67) | 14 (56.00) | 13 (72.22) |  |
| N1 | 166 (30.02) | 148 (29.72) | 5 (41.67) | 9 (36.00) | 4 (22.22) |  |
| N2 | 73 (13.20) | 68 (13.65) | 2 (16.67) | 2 (8.00) | 1 (5.56) |  |
| Lymph node yield, n (%) |  |  |  |  |  | 0.935 |
| <12 | 84 (15.19) | 76 (15.26) | 2 (16.67) | 3 (12.00) | 3 (16.67) |  |
| ≥12 | 469 (84.81) | 422 (84.74) | 10 (83.33) | 22 (88.00) | 15 (83.33) |  |
| Lymph vascular invasion, n (%) |  |  |  |  |  | 0.808 |
| Yes | 40 (7.23) | 37 (7.43) | 1 (8.33) | 0 (0.00) | 2 (11.11) |  |
| No | 79 (14.29) | 72 (14.46) | 1 (8.33) | 4 (16.00) | 2 (11.11) |  |
| Unknown | 434 (78.48) | 389 (78.11) | 10 (83.33) | 21 (84.00) | 14 (77.78) |  |
| Perineural invasion, n (%) |  |  |  |  |  | 0.983 |
| Yes | 8 (1.45) | 8 (1.61) | 0 (0.00) | 0 (0.00) | 0 (0.00) |  |
| No | 105 (18.99) | 95 (19.08) | 2 (16.67) | 4 (16.00) | 4 (22.22) |  |
| Unknown | 440 (79.57) | 395 (79.32) | 10 (83.33) | 21 (84.00) | 14 (77.78) |  |
| Tumor deposit, n (%) |  |  |  |  |  | 0.461 |
| Yes | 69 (12.48) | 62 (12.45) | 3 (25.00) | 3 (12.00) | 1 (5.56) |  |
| No | 484 (87.52) | 436 (87.55) | 9 (75.00) | 22 (88.00) | 17 (94.44) |  |
| Adjuvant chemotherapy, n (%) |  |  |  |  |  | 0.040 |
| Yes | 405 (73.24) | 365 (73.29) | 6 (50.00) | 17 (68.00) | 17 (94.44) |  |
| No | 148 (26.76) | 133 (26.71) | 6 (50.00) | 8 (32.00) | 1 (5.56) |  |

Note：

^1^n (%); Median (IQR)

^2^Pearson's Chi-squared test; Wilcoxon rank sum test; Fisher's exact test

Abbreviations: IQR, interquartile range; SD, standard deviation; COPD, Chronic Obstructive Pulmonary Disease; ECOG, Eastern Cooperative Oncology Group

# **Table S7. Demographic and Clinicopathological Characteristics Categorized by Skeletal Muscle Changes at postoperative 12 months**

| **Parameter** | **All Patients^1^**  **(n = 378)** | **Skeletal muscle changes at postoperative 6 months** | | | | ***P*-Value^2^** |
| --- | --- | --- | --- | --- | --- | --- |
|  |  | **High_pre_-High_post_^1^**  **(n = 341)** | **High_pre_-Low_post_^1^**  **(n = 11)** | **Low_pre_-High_post_^1^**  **(n = 11)** | **Low_pre_-Low_post_^1^**  **(n = 15)** |  |
| Age, year |  |  |  |  |  |  |
| Median (IQR) | 57.00 (49.25, 65.00) | 56.00 (49.00, 64.00) | 62.00 (58.00, 70.50) | 62.00 (50.00, 70.50) | 70.00 (67.00, 71.00) | <0.001 |
| Sex, n (%) |  |  |  |  |  | 0.143 |
| Female | 149 (39.42) | 138 (40.47) | 3 (27.27) | 1 (9.09) | 7 (46.67) |  |
| Male | 229 (60.58) | 203 (59.53) | 8 (72.73) | 10 (90.91) | 8 (53.33) |  |
| BMI, (kg/m2) |  |  |  |  |  |  |
| Median (IQR) | 23.03 (20.93, 25.34) | 23.31 (21.30, 25.43) | 21.91 (20.30, 24.74) | 20.58 (17.82, 21.12) | 20.76 (18.84, 20.78) | <0.001 |
| Smoking history, n (%) |  |  |  |  |  | 0.102 |
| Yes | 106 (28.04) | 94 (27.57) | 2 (18.18) | 6 (54.55) | 4 (26.67) |  |
| No | 268 (70.90) | 244 (71.55) | 9 (81.82) | 4 (36.36) | 11 (73.33) |  |
| Unknow | 4 (1.06) | 3 (0.88) | 0 (0.00) | 1 (9.09) | 0 (0.00) |  |
| Drinking history, n (%) |  |  |  |  |  | 0.808 |
| Yes | 86 (22.75) | 78 (22.87) | 2 (18.18) | 2 (18.18) | 4 (26.67) |  |
| No | 281 (74.34) | 253 (74.19) | 9 (81.82) | 8 (72.73) | 11 (73.33) |  |
| Unknow | 11 (2.91) | 10 (2.93) | 0 (0.00) | 1 (9.09) | 0 (0.00) |  |
| Hypertension, n (%) |  |  |  |  |  | 0.215 |
| Yes | 98 (25.93) | 88 (25.81) | 4 (36.36) | 2 (18.18) | 4 (26.67) |  |
| No | 278 (73.54) | 252 (73.90) | 7 (63.64) | 8 (72.73) | 11 (73.33) |  |
| Unknow | 2 (0.53) | 1 (0.29) | 0 (0.00) | 1 (9.09) | 0 (0.00) |  |
| Diabetes, n (%) |  |  |  |  |  | 0.013 |
| Yes | 30 (7.94) | 27 (7.92) | 3 (27.27) | 0 (0.00) | 0 (0.00) |  |
| No | 347 (91.80) | 314 (92.08) | 8 (72.73) | 10 (90.91) | 15 (100.00) |  |
| Unknow | 1 (0.26) | 0 (0.00) | 0 (0.00) | 1 (9.09) | 0 (0.00) |  |
| Coronary heart disease, n (%) |  |  |  |  |  | 0.047 |
| Yes | 15 (3.97) | 13 (3.81) | 0 (0.00) | 1 (9.09) | 1 (6.67) |  |
| No | 362 (95.77) | 328 (96.19) | 11 (100.00) | 9 (81.82) | 14 (93.33) |  |
| Unknow | 1 (0.26) | 0 (0.00) | 0 (0.00) | 1 (9.09) | 0 (0.00) |  |
| COPD, n (%) |  |  |  |  |  | 0.005 |
| Yes | 3 (0.79) | 1 (0.29) | 0 (0.00) | 0 (0.00) | 2 (13.33) |  |
| No | 372 (98.41) | 338 (99.12) | 11 (100.00) | 10 (90.91) | 13 (86.67) |  |
| Unknow | 3 (0.79) | 2 (0.59) | 0 (0.00) | 1 (9.09) | 0 (0.00) |  |
| ECOG grade, n (%) |  |  |  |  |  | 0.200 |
| 0 | 200 (52.91) | 186 (54.55) | 3 (27.27) | 5 (45.45) | 6 (40.00) |  |
| 1 | 173 (45.77) | 151 (44.28) | 8 (72.73) | 6 (54.55) | 8 (53.33) |  |
| 2 | 3 (0.79) | 2 (0.59) | 0 (0.00) | 0 (0.00) | 1 (6.67) |  |
| ≥3 | 2 (0.53) | 2 (0.59) | 0 (0.00) | 0 (0.00) | 0 (0.00) |  |
| Weight change at postoperative 3 months, (kg) | |  |  |  |  |  |
| Median (IQR) | -3.00 (-5.00, -1.00) | -3.00 (-5.00, -1.00) | -6.00 (-8.00, -4.50) | -2.00 (-4.00, -1.00) | -1.25 (-4.75, -0.25) | 0.034 |
| Weight change at postoperative 3 months, n (%) | |  |  |  |  | 0.672 |
| Decline | 252 (66.67) | 225 (65.98) | 10 (90.91) | 7 (63.64) | 10 (66.67) |  |
| Increase | 69 (18.25) | 62 (18.18) | 1 (9.09) | 2 (18.18) | 4 (26.67) |  |
| Unknown | 57 (15.08) | 54 (15.84) | 0 (0.00) | 2 (18.18) | 1 (6.67) |  |
| Preoperative serum albumin, (g/L) |  |  |  |  |  |  |
| Median (IQR) | 46.00 (43.75, 48.00) | 46.00 (44.00, 48.00) | 46.00 (46.00, 47.50) | 48.00 (43.50, 50.00) | 46.00 (41.50, 49.00) | 0.733 |
| Preoperative serum albumin group, n (%) | |  |  |  |  | 0.608 |
| <35 | 7 (1.85) | 6 (1.76) | 0 (0.00) | 0 (0.00) | 1 (6.67) |  |
| ≥35 | 369 (97.62) | 333 (97.65) | 11 (100.00) | 11 (100.00) | 14 (93.33) |  |
| Unknown | 2 (0.53) | 2 (0.59) | 0 (0.00) | 0 (0.00) | 0 (0.00) |  |
| Primary site, n (%) |  |  |  |  |  | 0.462 |
| Colon | 176 (46.56) | 163 (47.80) | 3 (27.27) | 4 (36.36) | 6 (40.00) |  |
| Rectum | 202 (53.44) | 178 (52.20) | 8 (72.73) | 7 (63.64) | 9 (60.00) |  |
| Pathological stage, n (%) |  |  |  |  |  | 0.267 |
| I | 84 (22.22) | 78 (22.87) | 0 (0.00) | 4 (36.36) | 2 (13.33) |  |
| II | 146 (38.62) | 130 (38.12) | 4 (36.36) | 5 (45.45) | 7 (46.67) |  |
| III | 148 (39.15) | 133 (39.00) | 7 (63.64) | 2 (18.18) | 6 (40.00) |  |
| Tumor differentiation, n (%) |  |  |  |  |  | 0.832 |
| Well+ Moderate | 100 (26.46) | 89 (26.10) | 2 (18.18) | 3 (27.27) | 6 (40.00) |  |
| Poor | 253 (66.93) | 228 (66.86) | 8 (72.73) | 8 (72.73) | 9 (60.00) |  |
| Unknown | 25 (6.61) | 24 (7.04) | 1 (9.09) | 0 (0.00) | 0 (0.00) |  |
| Histologic type, n (%) |  |  |  |  |  | 0.751 |
| Mucinous type | 350 (92.59) | 313 (91.79) | 11 (100.00) | 11 (100.00) | 15 (100.00) |  |
| Non–Mucinous type | 28 (7.41) | 28 (8.21) | 0 (0.00) | 0 (0.00) | 0 (0.00) |  |
| T stage, n (%) |  |  |  |  |  | 0.581 |
| T1 | 29 (7.67) | 27 (7.92) | 0 (0.00) | 1 (9.09) | 1 (6.67) |  |
| T2 | 67 (17.72) | 63 (18.48) | 0 (0.00) | 3 (27.27) | 1 (6.67) |  |
| T3 | 265 (70.11) | 235 (68.91) | 10 (90.91) | 7 (63.64) | 13 (86.67) |  |
| T4 | 17 (4.50) | 16 (4.69) | 1 (9.09) | 0 (0.00) | 0 (0.00) |  |
| N stage, n (%) |  |  |  |  |  | 0.263 |
| N0 | 228 (60.32) | 207 (60.70) | 4 (36.36) | 9 (81.82) | 8 (53.33) |  |
| N1 | 114 (30.16) | 103 (30.21) | 4 (36.36) | 2 (18.18) | 5 (33.33) |  |
| N2 | 36 (9.52) | 31 (9.09) | 3 (27.27) | 0 (0.00) | 2 (13.33) |  |
| Lymph node yield, n (%) |  |  |  |  |  | 0.498 |
| <12 | 54 (14.29) | 47 (13.78) | 2 (18.18) | 3 (27.27) | 2 (13.33) |  |
| ≥12 | 324 (85.71) | 294 (86.22) | 9 (81.82) | 8 (72.73) | 13 (86.67) |  |
| Lymph vascular invasion, n (%) |  |  |  |  |  | 0.913 |
| Yes | 43 (11.38) | 41 (12.02) | 1 (9.09) | 0 (0.00) | 1 (6.67) |  |
| No | 52 (13.76) | 48 (14.08) | 2 (18.18) | 1 (9.09) | 1 (6.67) |  |
| Unknown | 283 (74.87) | 252 (73.90) | 8 (72.73) | 10 (90.91) | 13 (86.67) |  |
| Perineural invasion, n (%) |  |  |  |  |  | 0.892 |
| Yes | 10 (2.65) | 10 (2.93) | 0 (0.00) | 0 (0.00) | 0 (0.00) |  |
| No | 76 (20.11) | 70 (20.53) | 3 (27.27) | 1 (9.09) | 2 (13.33) |  |
| Unknown | 292 (77.25) | 261 (76.54) | 8 (72.73) | 10 (90.91) | 13 (86.67) |  |
| Tumor deposit, n (%) |  |  |  |  |  | 0.591 |
| Yes | 40 (10.58) | 37 (10.85) | 2 (18.18) | 0 (0.00) | 1 (6.67) |  |
| No | 338 (89.42) | 304 (89.15) | 9 (81.82) | 11 (100.00) | 14 (93.33) |  |
| Adjuvant chemotherapy, n (%) |  |  |  |  |  | 0.077 |
| Yes | 266 (70.37) | 241 (70.67) | 9 (81.82) | 4 (36.36) | 12 (80.00) |  |
| No | 112 (29.63) | 100 (29.33) | 2 (18.18) | 7 (63.64) | 3 (20.00) |  |

Note：

^1^n (%); Median (IQR)

^2^Pearson's Chi-squared test; Wilcoxon rank sum test; Fisher's exact test

Abbreviations: IQR, interquartile range; SD, standard deviation; COPD, Chronic Obstructive Pulmonary Disease; ECOG, Eastern Cooperative Oncology Group

# **Table S8.** **Univariate and multivariate analysis of skeletal muscle index and recurrence free survival at preoperative baseline, postoperative 3, 6, 9, and 12 months.**

| **Variable** | **Univariate analysis** | | |  | **Multivariate analysis (M1)** | |  | **Multivariate analysis (M2)** | |
| --- | --- | --- | --- | --- | --- | --- | --- | --- | --- |
|  | **n (%)** | **HR (95%CI)** | ***P* value** |  | **HR (95%CI)** | ***P* value** | | **HR (95%CI)** | ***P* value** |
| **Preoperative baseline** |  |  |  |  |  |  |  |  |  |
| SMI normal group | 2000 (90.01) | Ref |  |  | Ref |  |  | Ref |  |
| Sarcopenia group | 222 (9.99) | 1.28 (1.01, 1.64) | 0.044 |  | 1.29 (1.00, 1.66) | 0.049 |  | 1.33 (1.03, 1.71) | 0.028 |
| **Postoperative 3 months** | |  |  |  |  |  |  |  |  |
| SMI normal group | 900 (93.07) | Ref |  |  | Ref |  |  | Ref |  |
| Sarcopenia group | 67 (6.93) | 1.27 (0.79, 2.06) | 0.329 |  | 1.29 (0.79, 2.11) | 0.304 |  | 1.38 (0.84, 2.26) | 0.204 |
| **Postoperative 6 months** | |  |  |  |  |  |  |  |  |
| SMI normal group | 662 (93.50) | Ref |  |  | Ref |  |  | Ref |  |
| Sarcopenia group | 46 (6.50) | 1.69 (1.03, 2.78) | 0.040 |  | 1.70 (1.01, 2.86) | 0.045 |  | 2.10 (1.23, 3.60) | 0.007 |
| **Postoperative 9 months** | |  |  |  |  |  |  |  |  |
| SMI normal group | 523 (94.58) | Ref |  |  | Ref |  |  | Ref |  |
| Sarcopenia group | 30 (5.42) | 1.88 (1.02, 3.49) | 0.044 |  | 1.86 (0.98, 3.51) | 0.057 |  | 2.06 (1.07, 3.96) | 0.030 |
| **Postoperative 12 months** | |  |  |  |  |  |  |  |  |
| SMI normal group | 352 (93.12) | Ref |  |  | Ref |  |  | Ref |  |
| Sarcopenia group | 26 (6.88) | 2.69 (1.49, 4.84) | <0.001 |  | 2.62 (1.34, 5.12) | 0.005 |  | 2.35 (1.18, 4.67) | 0.015 |

Note:

^1^Multivariate analysis (M1) was adjusted for sex, age (continuous), Smoking history, Drinking history, Hypertension, Diabetes, Coronary heart disease, COPD and ECOG grade;

^2^Multivariate analysis (M2) was adjusted for multivariate analysis (M1) plus weight change at postoperative 3 months, preoperative serum albumin (binary), primary site, pathological stage, lymph node yield, tumor differentiation, histologic type, perineural invasion, lymph vascular invasion, tumor deposit, adjuvant chemotherapy.

Abbreviations: CI, confidence interval; HR, hazard ratio; Ref, reference.

# **Table S9.** **Univariate and multivariate analysis of skeletal muscle index and overall survival at preoperative baseline, postoperative 3, 6, 9, and 12 months.**

| **Variable** | **Univariate analysis** | | |  | **Multivariate analysis (M1)** | |  | **Multivariate analysis (M2)** | |
| --- | --- | --- | --- | --- | --- | --- | --- | --- | --- |
|  | **n (%)** | **HR (95%CI)** | ***P* value** |  | **HR (95%CI)** | ***P* value** | | **HR (95%CI)** | ***P* value** |
| **Preoperative baseline** |  |  |  |  |  |  |  |  |  |
| SMI normal group | 2000 (90.01) | Ref |  |  | Ref |  |  | Ref |  |
| Sarcopenia group | 222 (9.99) | 1.41 (1.05, 1.91) | 0.022 |  | 1.3 (0.95, 1.77) | 0.096 |  | 1.32 (0.96, 1.80) | 0.086 |
| **Postoperative 3 months** | |  |  |  |  |  |  |  |  |
| SMI normal group | 900 (93.07) | Ref |  |  | Ref |  |  | Ref |  |
| Sarcopenia group | 67 (6.93) | 1.35 (0.68, 2.67) | 0.390 |  | 1.3 (0.65, 2.59) | 0.464 |  | 1.52 (0.75, 3.09) | 0.246 |
| **Postoperative 6 months** | |  |  |  |  |  |  |  |  |
| SMI normal group | 662 (93.50) | Ref |  |  | Ref |  |  | Ref |  |
| Sarcopenia group | 46 (6.50) | 2.20 (1.14, 4.26) | 0.019 |  | 2.12 (1.07, 4.19) | 0.031 |  | 2.82 (1.38, 5.77) | 0.005 |
| **Postoperative 9 months** | |  |  |  |  |  |  |  |  |
| SMI normal group | 523 (94.58) | Ref |  |  | Ref |  |  | Ref |  |
| Sarcopenia group | 30 (5.42) | 2.35 (1.01, 5.48) | 0.048 |  | 2.22 (0.92, 5.34) | 0.075 |  | 1.87 (0.75, 4.69) | 0.183 |
| **Postoperative 12 months** | |  |  |  |  |  |  |  |  |
| SMI normal group | 352 (93.12) | Ref |  |  | Ref |  |  | Ref |  |
| Sarcopenia group | 26 (6.88) | 4.06 (1.94, 8.50) | <0.001 |  | 4.71 (2.1, 10.54) | <0.001 |  | 4.28 (1.74, 10.52) | 0.002 |

Note:

^1^Multivariate analysis (M1) was adjusted for sex, age (continuous), Smoking history, Drinking history, Hypertension, Diabetes, Coronary heart disease, COPD and ECOG grade;

^2^Multivariate analysis (M2) was adjusted for multivariate analysis (M1) plus weight change at postoperative 3 months, preoperative serum albumin (binary), primary site, pathological stage, lymph node yield, tumor differentiation, histologic type, perineural invasion, lymph vascular invasion, tumor deposit, adjuvant chemotherapy.

Abbreviations: CI, confidence interval; HR, hazard ratio; Ref, reference.
